# Oxidative Phosphorylation Is Required for Powering Motility and Development of the Sleeping Sickness Parasite Trypanosoma brucei in the Tsetse Fly Vector

**DOI:** 10.1128/mbio.02357-21

**Published:** 2022-01-11

**Authors:** Caroline E. Dewar, Aitor Casas-Sanchez, Constentin Dieme, Aline Crouzols, Lee R. Haines, Álvaro Acosta-Serrano, Brice Rotureau, Achim Schnaufer

**Affiliations:** a Institute of Immunology and Infection Research, University of Edinburgh, Edinburgh, United Kingdom; b Department of Vector Biology, Liverpool School of Tropical Medicine, Liverpool, United Kingdom; c Trypanosome Transmission Group, Trypanosome Cell Biology Unit, Institut Pasteur and INSERM U1201, Paris, France; d Department of Tropical Disease Biology, Liverpool School of Tropical Medicine, Liverpool, United Kingdom; University of Georgia

**Keywords:** ATP synthase, *Trypanosoma brucei*, human African trypanosomiasis, mitochondria, mitochondrial metabolism, oxidative phosphorylation, sleeping sickness, trypanosomes, tsetse fly

## Abstract

The single-celled parasite Trypanosoma brucei is transmitted by hematophagous tsetse flies. Life cycle progression from mammalian bloodstream form to tsetse midgut form and, subsequently, infective salivary gland form depends on complex developmental steps and migration within different fly tissues. As the parasite colonizes the glucose-poor insect midgut, ATP production is thought to depend on activation of mitochondrial amino acid catabolism via oxidative phosphorylation (OXPHOS). This process involves respiratory chain complexes and F_1_F_o_-ATP synthase and requires protein subunits of these complexes that are encoded in the parasite's mitochondrial DNA (kDNA). Here, we show that progressive loss of kDNA-encoded functions correlates with a decreasing ability to initiate and complete development in the tsetse. First, parasites with a mutated F_1_F_o_-ATP synthase with reduced capacity for OXPHOS can initiate differentiation from bloodstream to insect form, but they are unable to proliferate *in vitro*. Unexpectedly, these cells can still colonize the tsetse midgut. However, these parasites exhibit a motility defect and are severely impaired in colonizing or migrating to subsequent tsetse tissues. Second, parasites with a fully disrupted F_1_F_o_-ATP synthase complex that is completely unable to produce ATP by OXPHOS can still differentiate to the first insect stage *in vitro* but die within a few days and cannot establish a midgut infection *in vivo*. Third, parasites lacking kDNA entirely can initiate differentiation but die soon after. Together, these scenarios suggest that efficient ATP production via OXPHOS is not essential for initial colonization of the tsetse vector but is required to power trypanosome migration within the fly.

## INTRODUCTION

The protist parasite Trypanosoma brucei is the causative agent of human African trypanosomiasis, also called sleeping sickness (1). The life cycle of T. brucei is complicated in that it involves at least nine distinct stages within the mammalian bloodstream and tissues and the tsetse fly vector (2–4). The distinct biology of these life cycle stages is closely linked to changes in energy metabolism and regulation of the activity of respiratory chain and F_1_F_o_-ATP synthase complexes in the inner membrane of the parasite’s single mitochondrion. As in other eukaryotes, these complexes are composed of both nuclearly and mitochondrially encoded subunits, and regulation of their expression therefore requires tight coordination between these organelles. In kinetoplastids such as T. brucei, structure and expression of the mitochondrial genome, termed kinetoplast DNA (kDNA), are extraordinarily complex. T. brucei kDNA comprises dozens of maxicircles (the equivalent of mitochondrial DNA in other eukaryotes) and thousands of minicircles (5). The latter encode guide RNAs (gRNAs) for remodeling of 11 of the 18 maxicircle-encoded mRNAs by uridylyl insertion and deletion, a maturation process called RNA editing (6).

In the proliferative long slender bloodstream form (BSF), ATP production is nonmitochondrial and occurs via glycolysis, exploiting the glucose-rich environment within the mammalian bloodstream (7, 8). The BSF mitochondrion lacks cyanide‐sensitive oxygen uptake: respiratory complexes III (ubiquinol:cytochrome c oxidoreductase) and IV (cytochrome oxidase) are absent, and the corresponding maxicircle- and nucleus-encoded transcripts are present at only very low levels (9–13). The BSF mitochondrion also lacks or has less developed cristae, which are invaginations of the inner mitochondrial membrane that are ultrastructural hallmarks of oxidative phosphorylation (OXPHOS) (14, 15). Despite the absence of cytochrome-mediated respiration, BSF parasites are dependent on oxygen for survival, since an alternative oxidase (AOX) in the inner mitochondrial membrane is critically involved in maintaining the redox balance for glycolysis (16). BSF cells use the F_1_F_o_-ATP synthase complex in reverse: ATP hydrolysis by the F_1_ part provides the energy for pumping protons from the mitochondrial matrix through the F_o_ part into the intermembrane space. This activity generates a mitochondrial membrane potential (ΔΨm), which is essential for protein import and exchange of metabolites and therefore fundamental for mitochondrial function (17–19).

Long slender BSF T. brucei organisms differentiate to the cell cycle-arrested stumpy BSF upon reaching high parasite numbers, induced by quorum sensing (20–22). The stumpy form is preadapted to survival in the tsetse fly vector by being more resistant to pH and proteolytic stresses and by beginning to prepare its mitochondrial energy metabolism for the change in nutrient availability. This adaptation includes upregulation of mitochondrial ATP production capability via substrate-level phosphorylation and beginning of cristae formation (20, 23–25).

Once ingested with a blood meal, stumpy forms differentiate into proliferative procyclic forms (PCF) within the fly midgut. Mitochondrial ATP production is vital to the PCF (26–28). Its inner mitochondrial membrane is now invaginated to form prominent cristae and contains complexes I (NADH:ubiquinone oxidoreductase), II (succinate dehydrogenase), III, and IV of a classical cytochrome‐mediated respiratory chain, along with complex V (F_1_F_o_-ATP synthase) (29, 30). In the tsetse fly midgut, proline and intermediates of the tricarboxylic acid cycle are thought to be the main energy sources (31–35), with glucose being only transiently abundant after blood meals (36). Under glucose-depleted *in vitro* growth conditions, which are similar to the tsetse fly midgut environment, ATP production via OXPHOS is essential (28, 37). Under glucose-rich *in vitro* growth conditions, only ATP production by mitochondrial substrate-level phosphorylation appears to be essential in PCF (26, 27); however, the electron transport chain remains essential under these conditions (27, 38). Complexes III and IV, despite being structurally divergent (9, 39–43), are thought to have classical enzymatic roles in the PCF respiratory chain: by pumping protons from the mitochondrial matrix into the intermembrane space, they generate a proton motive force and a ΔΨm upon the uptake of oxygen (44, 45). Under glucose-rich conditions, it is possible that this generation of ΔΨm becomes the sole essential function of complexes III and IV. Regardless of whether production of mitochondrial ATP involves OXPHOS or substrate-level phosphorylation, it requires the ADP/ATP carrier (AAC) to function in the direction of ATP export from the mitochondria to fuel processes in other parts of the cell (46).

By around day 3 to 7 postinfection, PCF are found in the ectoperitrophic space of the midgut, which, according to a long-standing model, they have reached by crossing the peritrophic matrix (PM) in the anterior midgut (4). However, recent findings suggest that T. brucei colonizes the proventriculus’ (PV) ectoperitrophic space by crossing the immature, fluid-like PM before it hardens (47). Within this revisited model, procyclic trypanosomes may colonize first the anterior midgut or establish parallel infections at both the anterior midgut and PV (47–51). Regardless of the topology of the infection, after approximately 2 weeks, parasites differentiate into long epimastigotes within the PV lumen, a process that involves the reversal of the positions of the kinetoplast and nucleus (52). Long epimastigotes then divide asymmetrically into long and short epimastigotes, aiding the delivery of short, less motile epimastigotes to the salivary glands (53, 54). Few parasites are able to complete this complex migration (55), and only a small proportion (∼10%) of experimentally infected tsetse flies are found with infected salivary glands (56); in the field, salivary gland infection rates are even lower than experimentally obtained ones (<1%) (57–59). Once in the salivary glands, epimastigotes attach to the epithelium, proliferate, and thus colonize these tissues (4, 60). Epimastigotes undergo further asymmetric divisions to continuously yield trypomastigotes that are able to develop into cell cycle-arrested metacyclics around 3 to 4 weeks postinfection (61). After injection into the mammalian host upon a tsetse bite, metacyclics develop into BSFs to begin the life cycle again. Much less is known about potential changes in mitochondrial energy metabolism after the PCF parasites have left the midgut, owing to the difficulty of culturing other insect stages in the laboratory. Recently, it was discovered that overexpression of the regulatory RNA binding protein RBP6 in PCF triggers efficient differentiation into metacyclic forms *in vitro* (62). Using this experimental system, a switch from cytochrome-based to AOX-mediated respiration and a general retrogression of mitochondrial structure in metacyclic forms were demonstrated (62, 63).

The kDNA encodes essential components of the multisubunit complexes involved in OXPHOS or in the translation of these components: eight subunits of complex I (ND1 to -5 and ND7 to -9), subunit apocytochrome *b* of complex III, three subunits of complex IV (COXI to -III), subunit A6 of complex V, two hydrophobic protein subunits of the mitoribosome (S12 [RPS12] and S3 [MURF5]), and the two mitoribosomal rRNAs (64, 65). As discussed above, complex V is essential in both BSFs and PCF, and complexes III and IV are essential in PCF. As a consequence, mitochondrial gene expression is critical for survival of both BSF and PCF T. brucei, although with PCF, this has been formally assessed only *in vitro* (66–69). (The role of complex I in energy metabolism in either the BSF or PCF is uncertain; this has been debated extensively elsewhere [24, 70–77].)

Intriguingly, “akinetoplastic” or “dyskinetoplastic” BSF T. brucei cells, which lack all kDNA (kDNA^0^) or critical parts of kDNA (kDNA^−^), respectively, occur in nature as the subspecies Trypanosoma brucei *evansi* and Trypanosoma brucei *equiperdum* and have also been generated in the lab (64, 78–80). In nature, these kDNA mutants are directly transmitted from mammal to mammal, either by mechanical transfer via successive fly bites or sexually, i.e., without any cyclical development or proliferation in an insect vector. This apparent contradiction regarding the essentiality of kDNA in BSF T. brucei was resolved by the discovery of point mutations in the nucleus-encoded F_1_-ATPase subunit γ in akinetoplastic and dyskinetoplastic forms, for example, a change of leucine in position 262 to proline (L262P). When introduced into wild type BSFs, these mutations can compensate for kDNA loss by enabling cells to manufacture ΔΨm independently of the F_o_ part of the ATP synthase (17, 66, 81). Therefore, the F_o_ subunit A6 is no longer essential (and neither are, consequently, the mitoribosomal protein and rRNA subunits), and the cells can survive as BSFs without any kDNA gene products.

The molecular mechanism of the compensatory mutations in subunit γ is not fully understood, but they enable F_o_-independent generation of ΔΨm via the electrogenic action of AAC-mediated ATP^4−^/ADP^3−^ exchange across the inner mitochondrial membrane (17, 24, 66, 82). Kinetic studies with yeast expressing mutated γ subunits have demonstrated a lowered *K_m_* value for ATP in the F_1_-ATPase reaction, suggesting that the mutant complex has a higher affinity for its substrate (17, 83). More efficient F_1_-mediated ATP hydrolysis, perhaps in the vicinity of the AAC, might support this essential electrogenic ATP^4−^/ADP^3−^ exchange (17, 83).

On the other hand, the mutations appear to impair the function of the F_1_F_o_ enzyme when it is required to operate in the direction of ATP synthesis. Physiological and structural studies in yeast have indicated that similar mutations in the yeast ATP synthase γ subunit alter its interactions with the catalytic F_1_-β subunit by partially uncoupling the F_1_ moiety from the F_o_ moiety (84–89). In the presence of an active electron transport chain, impaired coupling of F_1_ and F_o_ action will result in some dissipation of ΔΨm and, consequently, diminished efficiency of the ATP synthesis reaction.

In this study, we investigated the consequences of compensatory F_1_-γ mutations and kDNA loss for life cycle progression of T. brucei in the tsetse fly vector. Available evidence points to kDNA being essential for PCF viability and fly transmissibility (90). RNA interference (RNAi) knockdown of genes required for kDNA replication and division was lethal in PCF *in vitro* (69, 91–93), and chemically induced kDNA^0^ PCF cells were also nonviable *in vitro* (68, 94). Similarly, from a mixed population of kDNA^+^ and kDNA^0^ parasites (the latter were obtained by treatment of kDNA^+^ parasites with acriflavine), only kDNA^+^ cells were able to establish a tsetse midgut infection (95). Finally, *T. b. evansi* and *T. b. equiperdum* isolates have been documented as being unable to transform into PCF *in vitro* (81, 96, 97). A caveat with these studies was that they had been performed either with dying parasites unable to survive as BSFs, or with “monomorphic” strains that were unable to develop into the differentiation competent stumpy forms. The ability of viable, “pleomorphic” kDNA^0^ strains to infect and potentially develop in the tsetse fly vector has never been formally assessed.

Here, we built on our development of a range of pleomorphic BSF mutants that represent various defects in the expression of kDNA-encoded functions (Table 1) to assess the role of kDNA in stumpy-to-PCF differentiation, in the proliferation of PCF *in vitro*, and in the completion of the parasitic life cycle in tsetse flies.

**TABLE 1:** Mutations affecting trypanosome mitochondrial functions that were assessed for their effects on tsetse infectivity in this study^*a*^

Gene and mutation (TriTrypDB gene ID)	Expected consequence of mutation(s)	References
kDNA^0^ (NA)	Complete absence of essential subunits of respiratory complexes I, III, IV, and of the F_o_ part of complex V. Note that kDNA^0^ can be tested only in combination with a compensatory mutation, in this study the F_1_-γ L262P.	GenBank entry MK584625; 66
F_1_ subunit γ (Tb927.10.180), heterozygous or homozygous L262P mutation (WT/L262Pγ; L262P/L262Pγ)	F_1_-ATPase reaction: appears to lower *K_m_* for ATP and increase rate of ATP hydrolysis; F_1_F_o_-ATP synthase reaction: (partial?) uncoupling of F_o_-γ rotation and F_1_ activity, resulting in complete or partial loss of OXPHOS in homozygous mutant	17, 66, 84, 85, 88, 89
F_o_ subunit Tb1 (Tb927.10.520), null mutant (Tb1^−^/Tb1^−^)	Loss of F_1_F_o-_ATP synthase complex, accumulation of F_1_, resulting in complete loss of OXPHOS	107, 109

a See Table S1 for details on the corresponding cell lines. NA, not applicable.

10.1128/mBio.02357-21.9 TABLE S1 Cell lines used in this study. Download Table S1, PDF file, 0.4 MB. Copyright © 2022 Dewar et al. 2022 Dewar et al. https://creativecommons.org/licenses/by/4.0/ This content is distributed under the terms of the Creative Commons Attribution 4.0 International license.

## RESULTS

### Ability of F_1_F_o_-ATP synthase mutants and kDNA^0^ cells to differentiate to PCF *in vitro*.

To investigate the requirement for functional kDNA and a fully functional F_1_F_o_-ATP synthase in the differentiation of T. brucei from stumpy BSF to PCF, we generated a set of mutant cell lines (Table 1; also, see Table S1 in the supplemental material). In the pleomorphic cell line EATRO 1125 (AnTat1.1 90:13), we first replaced one allele or both alleles of the nucleus-encoded F_1_F_o_-ATP synthase subunit γ with either a wild-type version (WTγ; serving as a control) or a version with the L262P mutation (L262Pγ), as detailed in reference 24. This resulted in the cell lines WT/WTγ, WT/L262Pγ, and L262P/L262Pγ, respectively (Table 1 and Table S1). The L262Pγ mutation, in a homozygous or heterozygous configuration, enables slender BSF T. brucei to survive and proliferate without kDNA (66). We generated two cell lines lacking kDNA (kDNA^0^ 1 and 2) by treating two distinct clones of genotype WT/L262Pγ (clones 1 and 2) with the kDNA-intercalating dye acriflavine (24). The absence of kDNA in these two cell lines and the lack of an *in vitro* growth phenotype in the absence of kDNA have been shown previously (24). Moreover, T. brucei kDNA^0^ parasites can still differentiate to the stumpy BSF (24).

**FIG 1:**
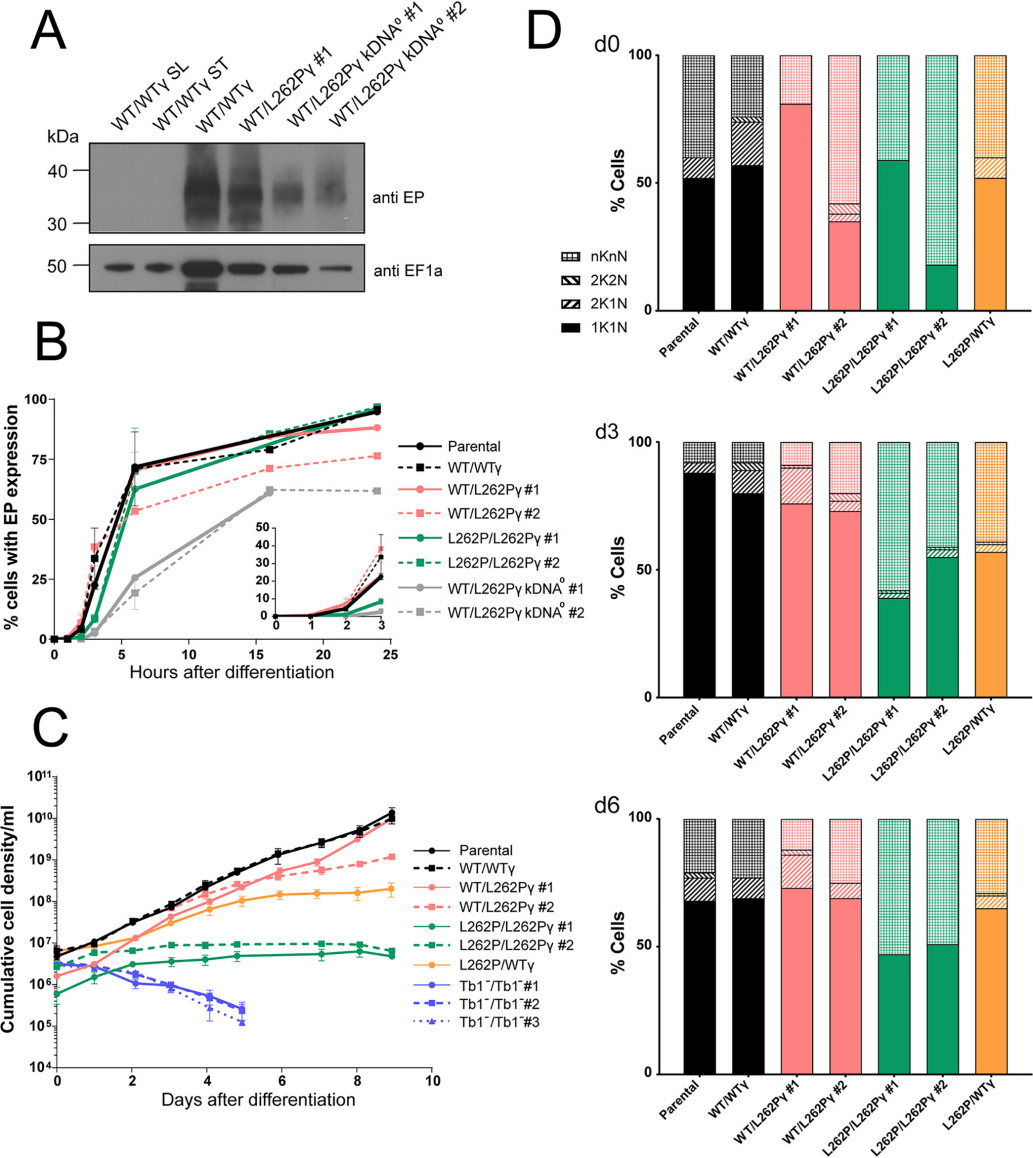
Ability of kDNA^0^ cells and F_1_F_o_-ATP synthase mutants to differentiate into PCF *in vitro*. (A) Western blot to assess EP procyclin expression. Stumpy cells were treated with 6 mM *cis*-aconitate (CA) and incubated at 27°C to induce differentiation. Cells were harvested after 24 h, 2 × 10^6^ cells were loaded per lane of an SDS-PAGE gel, and blots were probed with anti-EP procyclin (showing an expected smear due to heterogenous nature of EP protein [top]) and anti-EF1α as a loading control (bottom). Slender WT/WTγ cells and stumpy WT/WTγ cells not exposed to CA were analyzed as negative controls for EP expression. (B) EP expression measured by flow cytometry. Stumpy cells were treated with 6 mM CA and incubated at 27°C at 0 h. At each time point, cells were fixed and stained with mouse anti-EP procyclin and anti-mouse IgG Alexa 488 antibodies. Ten thousand cells were analyzed per sample; *n* = 2. (Inset) Magnification of the time points taken between 0 and 3 h after differentiation. Note that for kDNA^0^ cells, numbers at 24 h represent large amounts of cell debris that stained positive for EP. No intact, motile cells were visible upon inspection by microscopy at this time point. (C) Growth of newly differentiated PCF T. brucei. Stumpy form cells were treated with 6 mM CA in HMI-9 medium for 24 h at 27°C. Cells were transferred to SDM80 medium at 27°C (0 h), and cell density was determined with a hemocytometer over 24 h. Counting was performed in triplicate with three separate blood cryostocks of each cell line thawed and differentiated at the same time. Error bars indicate standard deviations. kDNA^0^ cells were not included in this analysis due to their death within 24 h after induction of differentiation. (D) Cell cycle analysis of newly differentiated PCF T. brucei. Slides were prepared at day 0 (d0), d3, and d6 after differentiation and transfer into SDM80, and cells were blindly scored for cell cycle stage. Approximately 100 cells were assessed per strain and time point. The category *n*K*n*N includes zoids (i.e., cells with no nucleus), monsters (multiple nuclei), and dying (large, blurry, and/or fragmented nuclei) cells. kDNA^0^ and Tb1^−^/Tb1^−^ cells were not included in this analysis due to their nonviability after differentiation.

To test the requirement of kDNA for differentiation to PCF, we harvested stumpy kDNA^0^ and control cells from infected mice and induced differentiation into PCF *in vitro* by adding 6 mM *cis*-aconitate (CA) to the medium and shifting the temperature from 37°C to 27°C for 24 h (98–102). By microscopic inspection, no intact, motile kDNA^0^ cells were visible at this point, with only nonmotile cells and debris remaining. This contrasted with all other cell lines, which remained motile, although some debris was present. We then harvested cultures by centrifugation and assessed lysates by Western blotting for expression of EP procyclin, an early marker for PCF differentiation (103–105) (Fig. 1A). Compared to WT/WTγ and WT/L262Pγ lysates, the EP procyclin signal was much reduced in material recovered from kDNA^0^ cultures. These results suggest that kDNA^0^ BSF cells differentiated to PCF but died soon afterward, which implies that the requirement for kDNA in PCF cells cannot be compensated for by the L262Pγ mutation.

We performed a time course to compare the kinetics of EP procyclin expression after induction of differentiation with CA in cells with and without kDNA. We collected samples from each cultured cell line at 0, 1, 2, 3, 6, 16, and 24 h after CA induction (+CA), stained them with mouse anti-EP procyclin antibody and anti-mouse IgG Alexa Fluor 488, and analyzed them by flow cytometry (Fig. 1B). Parental, WT/WTγ, and WT/L262Pγ cells containing kDNA expressed EP procyclin with similar kinetics; 55 to 70% of cells expressed EP procyclin by 6 h postinduction. Two clones (clones 1 and 2) of the L262P/L262Pγ cell line initially showed slower kinetics of EP procyclin expression (Fig. 1B, inset), but after 6 h, the percentage of EP procyclin-positive cells was similar to that in other cell lines with kDNA. kDNA^0^ cells, on the other hand, showed a delayed rate of EP procyclin expression, with the proportion of cells expressing the protein increasing to a maximum of 55% by 16 h. As the vast majority of kDNA^0^ cells had died by 24 h after CA induction, this figure included EP procyclin-positive cell debris, which was not gated out of this analysis.

To test if freshly differentiated PCF cells expressing one or two of the mutated L262Pγ alleles could reenter the cell cycle and proliferate under conditions that require efficient OXPHOS, we resuspended cells in SDM80 medium after 24 h of CA treatment. SDM80 lacks added glucose and contains *N*-acetyl d-glucosamine to inhibit uptake of residual glucose from fetal calf serum (FCS); it is in this low-glucose environment that PCF depend on OXPHOS to generate ATP (28, 37). Here, day 0 (d0) was defined as being after 24 h in HMI-9 at 27°C plus CA and at 24 h in SDM80 at 27°C. Under these conditions, freshly differentiated WT/WTγ and WT/L262Pγ cells were able to proliferate (Fig. 1C), and analysis of the number of kinetoplasts (K) and nuclei (N) per cell confirmed that they could progress through the cell cycle (Fig. 1D). Cells with two kinetoplasts and one nucleus (2K1N) or with two kinetoplasts and two nuclei (2K2N) were consistently seen in culture across the time course, indicating actively dividing cells. Homozygous L262P/L262Pγ cell lines, however, did not grow in this medium. Although these cells survived under these conditions, they did not thrive and merely maintained the population density. Observation by microscopy revealed that these cells were motile but that forward motility appeared to be impaired compared to that in the other cell lines (Movies S1 to S4). The L262P/L262Pγ cells were unable to progress through the cell cycle, with barely any 2K1N or 2K2N cell types detectable (Fig. 1D).

10.1128/mBio.02357-21.5 MOVIE S1 Microscopic video recording of cell line WT/WTγ taken at day 6 after differentiation. Due to the limit of the number of supplemental items allowed, recordings for only four cell lines could be provided. Download Movie S1, AVI file, 0.6 MB. Copyright © 2022 Dewar et al. 2022 Dewar et al. https://creativecommons.org/licenses/by/4.0/ This content is distributed under the terms of the Creative Commons Attribution 4.0 International license.

10.1128/mBio.02357-21.6 MOVIE S2 Microscopic video recording of cell line WT/L262Pγ clone 1 taken at day 6 after differentiation. Due to the limit of the number of supplemental items allowed, recordings for only four cell lines could be provided. Download Movie S2, AVI file, 1.0 MB. Copyright © 2022 Dewar et al. 2022 Dewar et al. https://creativecommons.org/licenses/by/4.0/ This content is distributed under the terms of the Creative Commons Attribution 4.0 International license.

10.1128/mBio.02357-21.7 MOVIE S3 Microscopic video recording of cell line L262P/L262Pγ clone 1 taken at day 6 after differentiation. Due to the limit of the number of supplemental items allowed, recordings for only four cell lines could be provided. Download Movie S3, AVI file, 0.6 MB. Copyright © 2022 Dewar et al. 2022 Dewar et al. https://creativecommons.org/licenses/by/4.0/ This content is distributed under the terms of the Creative Commons Attribution 4.0 International license.

10.1128/mBio.02357-21.8 MOVIE S4 Microscopic video recording of cell line L262P/WTγ taken at day 6 after differentiation. Due to the limit of the number of supplemental items allowed, recordings for only four cell lines could be provided. Download Movie S4, AVI file, 1.0 MB. Copyright © 2022 Dewar et al. 2022 Dewar et al. https://creativecommons.org/licenses/by/4.0/ This content is distributed under the terms of the Creative Commons Attribution 4.0 International license.

Cells in the category *n*K*n*N, which includes zoids (*n*K0N), “monsters” with more than two nuclei and/or kinetoplasts, and other dying cells, were visible in this analysis at every time point and with every cell genotype (Fig. 1D). This was possibly caused by cell swelling and/or nuclear fragmentation due to osmotic stress, which is a side effect of transferring freshly thawed stumpy cells from blood into liquid medium for the differentiation experiments. Notably, for the L262P/L262Pγ cell lines, the proportion of *n*K*n*N cells remained extremely high as the time course continued, consistent with the high proportion of dying cells in these populations.

To confirm that the L262P/L262Pγ genotype, and not a secondary deficiency, was responsible for the severe growth defect of these PCF cell lines, we generated an L262P/WTγ (WTγ add-back) cell line by replacing one subunit γ allele in cell line L262P/L262Pγ clone 2 with a WT subunit γ gene. The ability to maintain growth in SDM80 and progress through the cell cycle was partially restored in the resulting heterozygous γ cell line (Fig. 1C and D), thus confirming an effect specifically due to the L262Pγ mutation.

We then asked whether 10 mM glucose in the medium (SDM79 medium) could rescue the growth defect of homozygous L262Pγ PCF cells, as ATP production via OXPHOS was reported to be inessential under these conditions (26, 27, 106), although this may be cell line dependent (107). Surprisingly, we observed an immediate, severe growth phenotype in all freshly differentiated cell lines, with L262Pγ homozygotes having the strongest growth defect (Fig. S1A). In WT/WTγ or heterozygous WT/L262Pγ cells, this phenotype could be rescued by removal of glucose from the medium and restored by addition of glucose (Fig. S1B and C). This growth retardation was probably due to an impact of glucose signaling on metabolic adaptation in freshly differentiated PCF parasites (108).

10.1128/mBio.02357-21.1 FIG S1 Effect of glucose on growth rates of freshly differentiated PCF cells. (A) Cells were treated with 6 mM CA in HMI-9 for 24 h and then transferred to SDM80 medium supplemented with 10 mM glucose (solid lines) at a density of 2 × 10^6^/mL (day 0). An established PCF T. brucei cell line, 29:13, was used as control that grows in either SDM80 (residual glucose, dashed lines) or SDM80 supplemented with glucose. Over the next12 to 13 days, cells were counted once a day and afterwards were diluted to a density of at least 3 × 10^6^/mL. (B and C) After induction of differentiation with CA, WT/WTγ (B) and WT/L262Pγ clone 2 parasites (C) were initially grown in either SDM80 (− glucose) or SDM80 supplemented with 10 mM glucose (+ glucose). Red arrows depict transfer of cells from SDM80 with glucose into SDM80 minus glucose (+/− glucose), and blue arrows depict transfer of cells into SDM80 without glucose into SDM80 with glucose (−/+ glucose). Download FIG S1, PDF file, 0.7 MB. Copyright © 2022 Dewar et al. 2022 Dewar et al. https://creativecommons.org/licenses/by/4.0/ This content is distributed under the terms of the Creative Commons Attribution 4.0 International license.

Cells lacking kDNA and homozygous L262Pγ mutants differ in two key aspects: due to the absence of all kDNA-borne genes, the former cannot generate functional respiratory complexes III (cytochrome *bc*_1_ complex) and IV (cytochrome *c* oxidase) or the F_o_ part of the F_1_F_o_-ATP synthase. In the latter, the F_1_ and F_o_ parts are thought to be functionally uncoupled, although the extent of uncoupling is unclear (Table 1). To further investigate the basis for the phenotypic differences between these cell lines, we generated a cell line lacking ATP synthase subunit Tb1 (Tb1^−^/Tb1^−^) (Fig. S2). Tb1 (systematic TriTrypDB ID Tb927.10.520) is the largest T. brucei F_o_ subunit and required to maintain the intact F_1_F_o_ complex structure and ATP synthase activity; however, it is not required for F_1_-ATPase activity (107, 109). Like other F_o_ subunits, the gene becomes dispensable in BSF T. brucei in the presence of an L262Pγ allele, which enabled us to generate mutants that completely lack F_o_ but possess all kDNA-borne genes (Fig. S2). Three independent Tb1-null clones (Tb1^−^/Tb1^−^ clones 1, 2, and 3) were used in this study. This cell line was able to generate stumpy forms *in vivo*, but once differentiated to the PCF *in vitro*, they died rapidly (Fig. 1C). This suggests that ATP production by OXPHOS becomes critical upon differentiation into insect stage parasites and that the L262Pγ mutation only partially uncouples the F_1_F_o_-ATP synthase, permitting cell survival, if not proliferation (Fig. 1C), via residual OXPHOS activity.

10.1128/mBio.02357-21.2 FIG S2 Generation and verification of Tb1 null mutants. (A) Tb1 knockout plasmids produced by DNA synthesis (Biomatik). Drug selection markers (BSD, blasticidin resistance; PHL, phleomycin resistance) are flanked by actin 5′ and 3′ untranslated regions (UTR) for mRNA splicing and polyadenylation, respectively, and by Tb1 5′ and 3′ UTR or intergenic regions (IGR) for homologous recombination. (B) PCR verification of three Tb1^−^/Tb1^−^ clones (no. 1, 2, and 3). (Top) Duplex PCR with simultaneous amplification of an ∼700-bp fragment of the Tb1 coding sequence and an ∼900-bp fragment of the ATPase γ subunit gene as internal control. (Bottom) Amplification of the Tb1 locus using primers flanking the 5′ and 3′ recombination sites. The wild-type locus and the locus after replacement of the Tb1 coding sequence with the BSD and PHL genes give amplicons of 2,400 bp, 2,100 bp, and 1,540 bp, respectively. Genomic DNA from a WT/Tb1^−^ single-knockout cell line is included as a control. (C) Western blot verification of three Tb1^−^/Tb1^−^ clones, using a Tb1 antibody and an Hsp70 antibody as a control. Whole-cell lysates of 2 × 10^6^ cells were analyzed per lane. The asterisk indicates nonspecific detection of an ∼55-kDa protein by the Tb1 antibody. Lysates of an inducible Tb1 RNAi cell line (109), uninduced and induced for 4 days for Tb1 ablation with tetracycline (tet), are shown as a control. Download FIG S2, PDF file, 0.9 MB. Copyright © 2022 Dewar et al. 2022 Dewar et al. https://creativecommons.org/licenses/by/4.0/ This content is distributed under the terms of the Creative Commons Attribution 4.0 International license.

### Mitochondrial ATP production capacity in F_1_F_o_-ATP synthase mutants.

**FIG 2:**
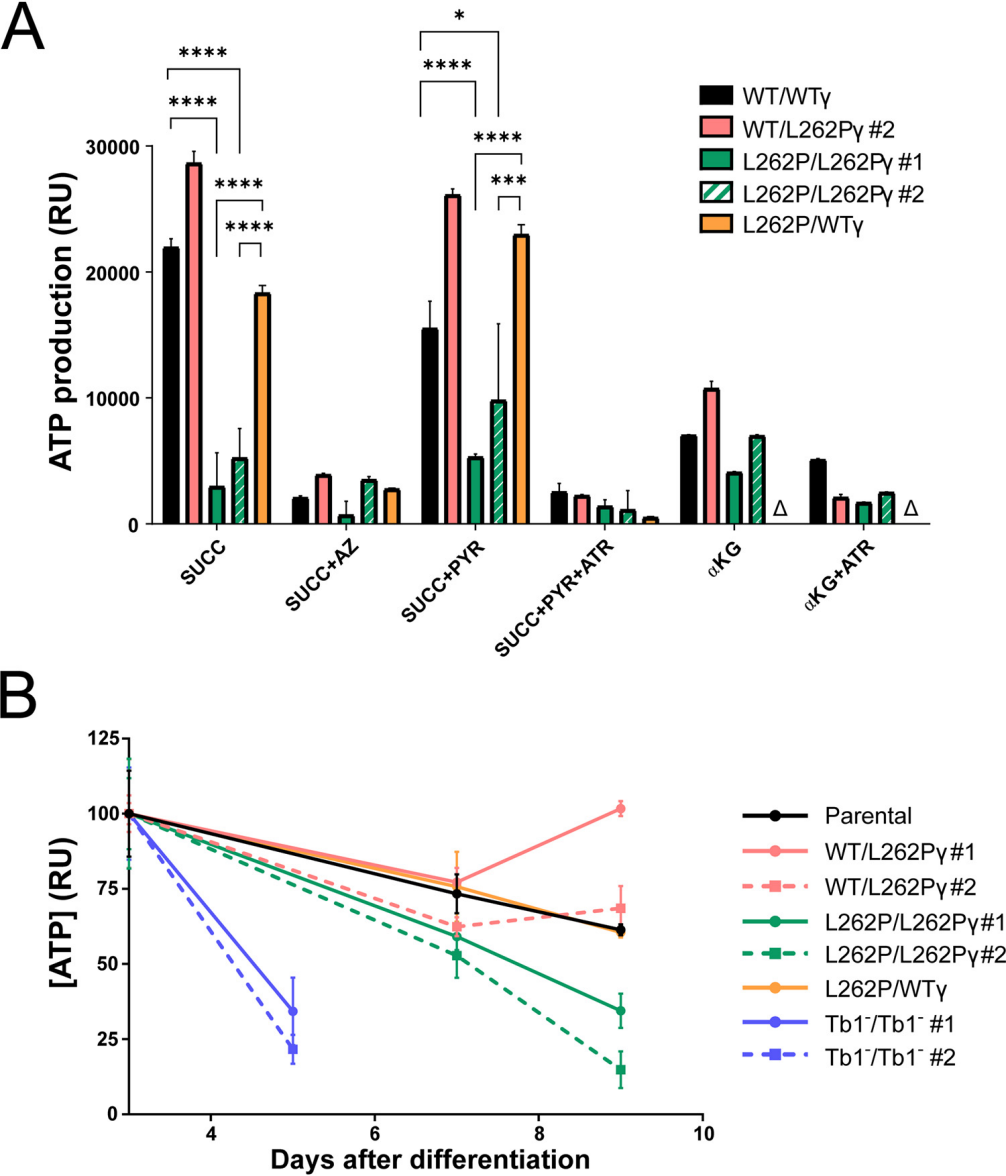
Mitochondrial ATP production capacity in F_1_F_o_-ATP synthase mutants. Cells were differentiated to PCF as described for Fig. 1 and maintained in SDM80 medium. (A) Mitochondrial ATP production assay using permeabilized differentiated PCF cells with intact mitochondria. The capacity for ATP production via OXPHOS (succinate [SUCC]), substrate-level phosphorylation via the acetate:succinate CoA transferase (ASCT)/succinyl-CoA synthetase (SCoAS) cycle (pyruvate [PYR]), and substrate-level phosphorylation via α-ketoglutarate dehydrogenase and SCoAS (αKG) was assessed. Azide (AZ) and atractyloside (ATR) are inhibitors of the F_1_F_o_-ATP synthase and AAC, respectively. All assays were performed in triplicate at day 2 after differentiation. Error bars indicate standard deviations. A triangle indicates that the assay was not performed. *P* values were determined using ANOVA: *, <0.05; **, <0.01; ***, <0.001; ****, <0.0001. See Table S2 for details of statistical comparisons. (B) Total cellular ATP level assays performed in triplicate, with the assay performed at days 3, 5, 7, and 9 post differentiation. The Tb1^−^/Tb1^−^ cell line was assayed only up to day 5, as insufficient numbers of viable cells remained after that time point. Error bars indicate standard deviations. The ATP level from day 3 for each cell line was set as 100%.

We next investigated the efficiency of mitochondrial ATP production pathways in the different cell lines. We added metabolic substrates that allowed ATP production by OXPHOS only (succinate) or a combination of OXPHOS and substrate phosphorylation (succinate plus pyruvate; α-ketoglutarate) to preparations of disrupted cells containing intact mitochondria. We then measured “*in organello*” ATP production via the generation of a proportional luminescent signal (110). Using succinate as a substrate, preparations from L262P/L262Pγ cells generated significantly less ATP than samples from cells with at least one WTγ allele (Fig. 2A and Table S2). Indeed, inhibition of the F_1_F_o_-ATP synthase with azide reduced ATP production in L262P/L262Pγ cell lysates only insignificantly, suggesting a very minor, if any, contribution to ATP production by the mutated enzyme under these conditions. Preparations of WT/L262Pγ cells produced higher ATP levels than those of WTγ cells; at present we have no explanation for this observation. The presence of one or two L262Pγ alleles did not affect the level of representative F_1_ and F_o_ ATP synthase subunits (Fig. S3), thus confirming the effect of the mutation is due to functional impairment. Adding back a WTγ allele to L262P/L262Pγ restored ATP production via OXPHOS nearly to levels found for WT/WTγ cells (Fig. 2A). Interestingly, adding pyruvate as an additional substrate, which theoretically should allow additional ATP production via the acetate:succinate coenzyme A (CoA) transferase (ASCT) cycle, did not significantly increase ATP production in mitochondria of any of the cell lines (Fig. 2A; Table S2). This suggests the absence or low levels of either important transporters, enzymes, or cofactors for this pathway in these early differentiated cells. Similarly, α-ketoglutarate as the sole substrate, which should allow ATP production by substrate phosphorylation via succinyl-CoA synthetase, with production of succinate and subsequent ATP production via OXPHOS, resulted in only a moderate production of ATP in mitochondria from newly differentiated WTγ cells.

10.1128/mBio.02357-21.3 FIG S3 Abundance of F_1_F_o_-ATP synthase in γ subunit mutants. Cellular levels of mitochondrial respiratory complexes F_1_F_o_-ATP synthase and cytochrome oxidase (COX) assessed by probing a Western blot with specific antibodies. Tb2 and β are subunits of the F_o_ and F_1_ moieties of the ATP synthase, respectively. COX is represented by subunits IV and VI. Cells were harvested after 48 h in SDM80 medium, and whole-cell lysates of 2 × 10^6^ cells were loaded per lane. Download FIG S3, PDF file, 0.8 MB. Copyright © 2022 Dewar et al. 2022 Dewar et al. https://creativecommons.org/licenses/by/4.0/ This content is distributed under the terms of the Creative Commons Attribution 4.0 International license.

10.1128/mBio.02357-21.10 TABLE S2 Statistical analysis by one-way ANOVA of selected data from Fig. 2A. Download Table S2, PDF file, 0.05 MB. Copyright © 2022 Dewar et al. 2022 Dewar et al. https://creativecommons.org/licenses/by/4.0/ This content is distributed under the terms of the Creative Commons Attribution 4.0 International license.

The maintenance of total cellular ATP levels after differentiation was also impacted by the presence of homozygous L262Pγ. By day 9, the level of cellular ATP in L262P/L262Pγ cells was considerably lower than that of cells expressing at least one WTγ allele (Fig. 2B). This is consistent with an impairment of ATP synthase complexes with an L262Pγ mutation in coupling the ΔΨm to ATP synthesis, as suggested above. ATP production assays could not be performed on Tb1^−^/Tb1^−^ cell lines due to the large number of viable differentiated cells required, but measurement of cellular ATP levels at day 5 showed that ATP levels were much depleted in these dying cells (Fig. 2B).

### Motility analysis of *in vitro* cultured F_1_F_o_-ATP synthase mutant cell lines.

**FIG 3:**
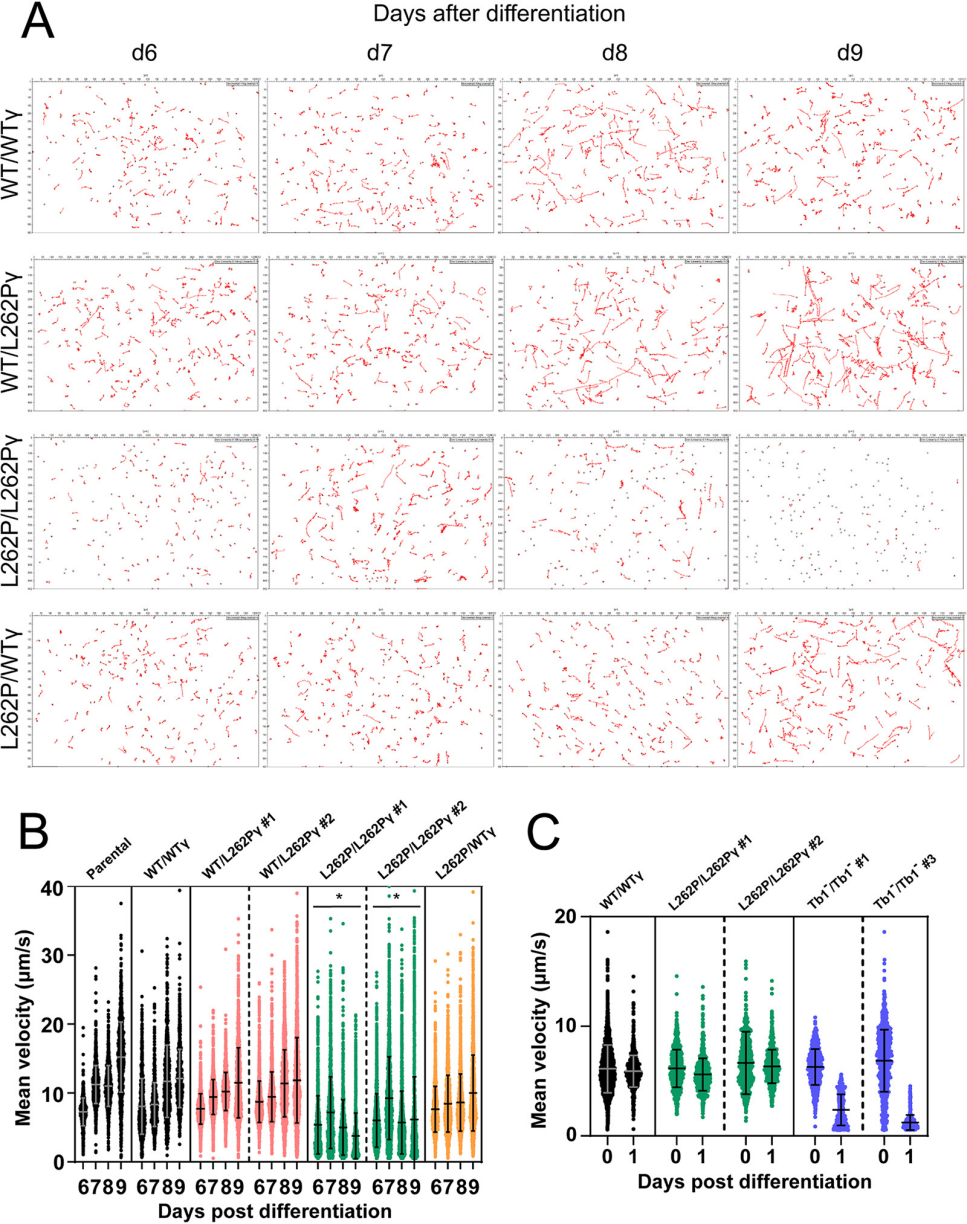
Motility analysis of *in vitro* cultured parasites. (A) Representative cell movement tracks taken from videos of differentiated PCF cells *in vitro*. Videos were taken of populations of newly differentiated PCF T. brucei at days 6, 7, 8, and 9 (d6 to d9) after differentiation. (B) Measurement of curvilinear velocity (VCL) from video tracking in panel A, expressed as mean velocity of each tracked cell. VCL was assessed at d6 to d9. At least 965 cells were tracked per cell line and time point. *P* values were determined using ANOVA between cell lines across the entire d6-d9 data set. *, *P* < 0.05 (significant reduction in motility in comparison to the other cell lines shown). Error bars indicate standard deviations. (C) VCL assessed as mean velocity of each tracked cell at d0 and d1. At least 750 cells were tracked per cell line and time point. Error bars indicate standard deviations. Representative tracks taken from videos of these cells are shown in Fig. S4.

Microscopic observation of L262P/L262Pγ cells grown in SDM80 medium had suggested a motility phenotype. We decided to investigate this further by recording motility tracks from videos of freshly differentiated cells in SDM80 medium. L262P/L262Pγ cells showed an evident motility defect in comparison with cells expressing at least one WTγ allele (Fig. 3A). The average curvilinear velocities measured over the actual point-to-point route followed by the cell, i.e., the mean instant speeds, were calculated from these tracks, which confirmed that L262P/L262Pγ cells had a substantial progressive velocity defect manifesting between day 6 and day 9 after differentiation (Fig. 3B). The WTγ add-back cell line L262P/WTγ, however, had a motility similar to that of WT/WTγ and WT/L262Pγ cells. An overall trend of increasing motility in the other cell lines between days 6 and 9, despite a trend of slightly declining total cellular ATP in at least the parental cell line (Fig. 2B), suggests that the relationship between total cellular ATP concentration and motility is not simply linear but that there is possibly a threshold of ATP availability below which motility cannot be maintained. Consistent with the more rapid drop in cellular ATP in Tb1^−^/Tb1^−^ cells, these mutants showed a pronounced reduction in mean velocity immediately after differentiation, at a time when the velocity of L262P/L262Pγ cells was still unaffected (Fig. 3C; Fig. S4). The rapid death of the Tb1^−^/Tb1^−^ cells after differentiation precluded an analysis of later time points.

10.1128/mBio.02357-21.4 FIG S4 Motility analysis of *in vitro* cultured cells. Representative cell movement tracks taken from videos of differentiated PCF cells *in vitro*. Videos were taken of populations of newly differentiated PCF T. brucei at d0 and d1 after differentiation, where d0 is defined as the time point after 24 h of exposure to 6 mM CA and 27°C and directly after transfer into SDM80 medium. Download FIG S4, PDF file, 1.2 MB. Copyright © 2022 Dewar et al. 2022 Dewar et al. https://creativecommons.org/licenses/by/4.0/ This content is distributed under the terms of the Creative Commons Attribution 4.0 International license.

### Ability of F_1_F_o_-ATP synthase mutants and kDNA^0^ cells to colonize the tsetse midgut.

**FIG 4:**
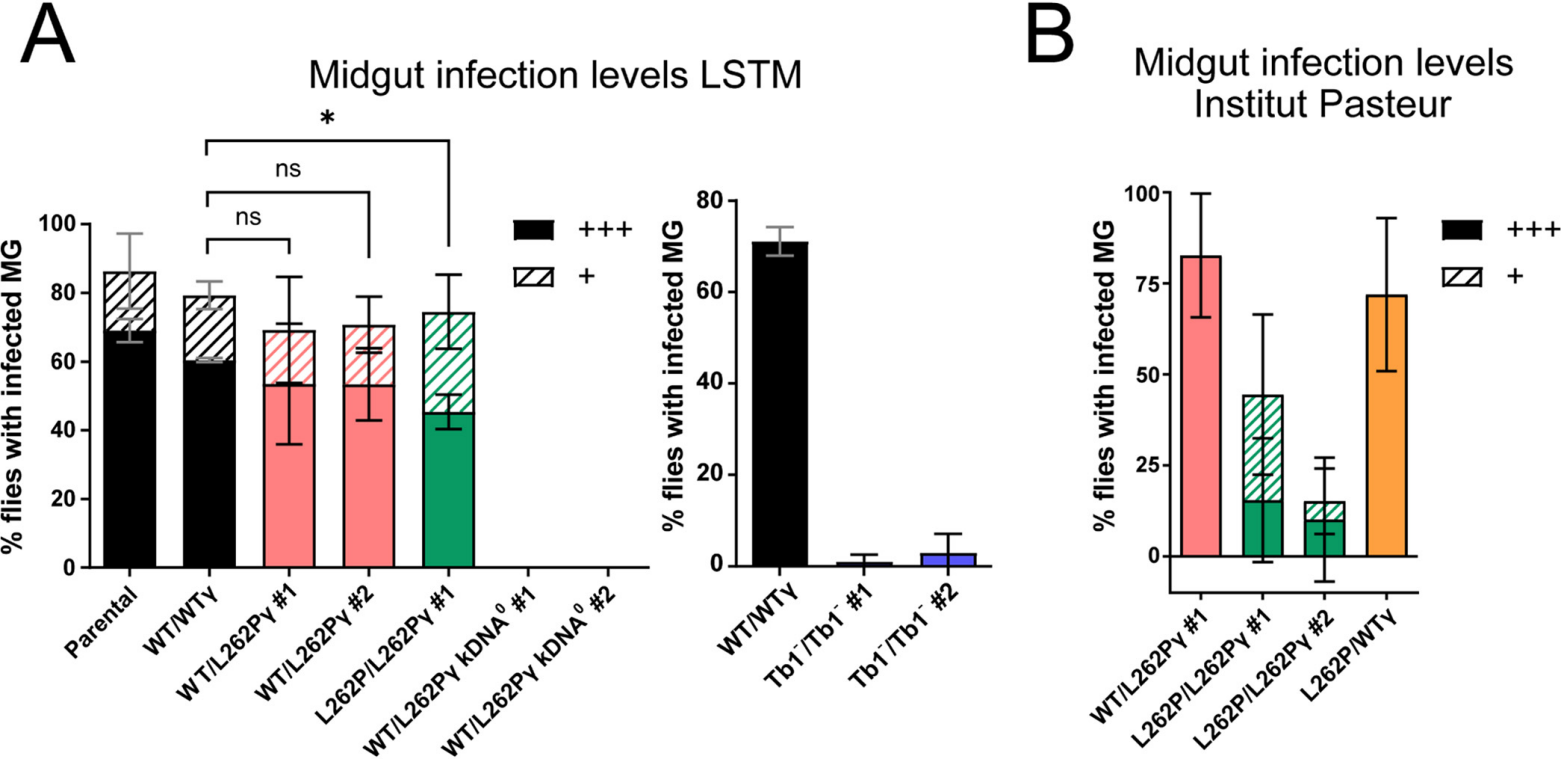
Assessment of fly midgut infectivity of parasites with ATP synthase and kDNA mutations. Midgut infection rates at tsetse fly facilities of (A) Liverpool School of Tropical Medicine (LSTM) and (B) Institut Pasteur Paris. Infected tsetse fly midguts (MG) were harvested at day 9 after the infected blood meal. Density of infection was judged by microscopy: +++, high level of midgut infection at >>10 parasites/field of view; +, low level of midgut infection at ∼10 parasites/field of view. For the experiment with Tb1^−^/Tb1^−^ cells and the corresponding WT/WTγ control (A, right), midguts were scored as infected if any trypanosomes were detected. Approximately 25 flies (or 50 flies for the Tb1^−^/Tb1^−^ cell lines and the corresponding control [A, right]) were infected with one blood cryostock of stumpy-form trypanosomes per cell line and replicate; *n* = 3. Error bars indicate standard deviations. *, *P* < 0.05, and ns, not significant, for comparison between the heavy infection levels (unpaired *t* test).

To test whether trypanosomes devoid of kDNA could establish a PCF midgut infection *in vivo*, we fed teneral tsetse flies with horse blood containing stumpy-form WT/L262Pγ kDNA^0^ parasites or kDNA^+^ stumpy-form parasites with the parental, WT/WTγ, WT/L262Pγ, or L262P/L262Pγ genotype at the Liverpool School of Tropical Medicine (LSTM) tsetse fly colony. At day 9 postinfection, we dissected the flies and isolated, disrupted, and inspected their midguts under a microscope to assess the parasite infection prevalence. The parental cell line was able to differentiate to the PCF and established a midgut infection in around 85% of flies dissected (Fig. 4A), with 70% of the flies having high levels of infection. In contrast, kDNA^0^ T. brucei cell lines were unable to establish even a single midgut infection.

Flies infected with WT/L262Pγ or L262P/L262Pγ cells had a slightly lower midgut infection level than flies infected with parental or WT/WTγ cells; approximately 70% were midgut infected, but the difference in overall infection rates was not statistically significant (Fig. 4A). However, flies infected with L262P/L262Pγ parasites showed a significantly lower proportion of highly infected midguts. Infections conducted at a second tsetse colony at Institut Pasteur, Paris, France, with a subset of the same cell lines showed more reduced levels of midgut infection for L262P/L262Pγ cells, and this phenotype was rescued in the WTγ add-back cell line (Fig. 4B). Flies infected in a separate experiment with Tb1^−^/Tb1^−^ cells produced infections that were barely detectable in the midgut (Fig. 4A), which is consistent with their rapid death after differentiation *in vitro* (Fig. 2B). When combined, these results demonstrate that T. brucei requires an F_1_F_o_-ATP synthase complex capable of functioning in OXPHOS to produce an efficient tsetse midgut infection. A reduced capacity for OXPHOS, as demonstrated by the homozygous L262Pγ parasites, can sustain a midgut infection, but at a reduced rate. Absence of functional F_1_F_o_ ATP synthase, as in Tb1^−^/Tb1^−^ cells, permits minimal midgut infection, whereas absence of all kDNA-encoded products completely prevents midgut infection.

### Ability of L262Pγ F_1_F_o_-ATP synthase mutants to colonize the tsetse fly PV.

**FIG 5:**
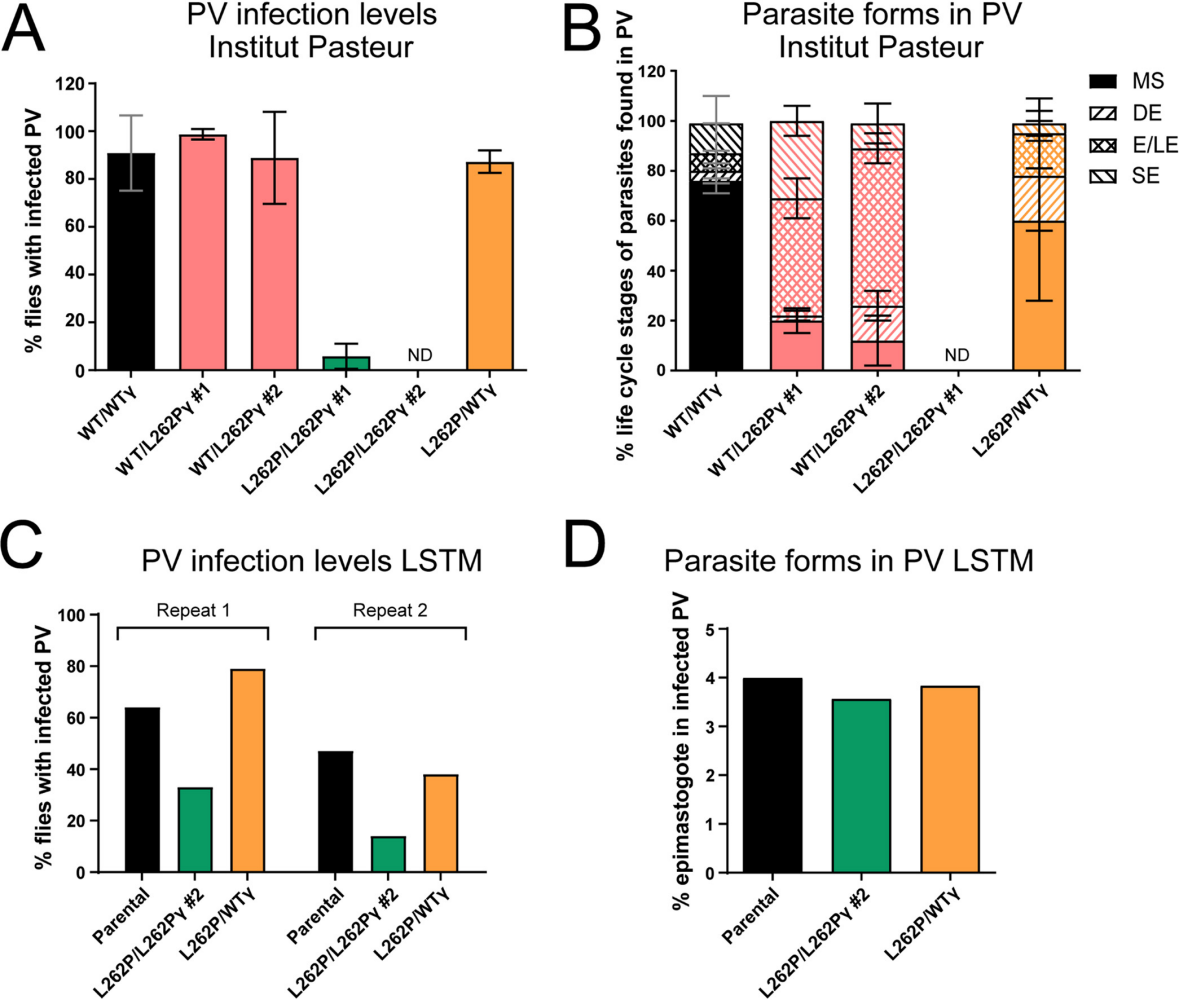
Assessment of fly PV infectivity of parasites with ATP synthase mutations. PV infection and parasite differentiation rates at the tsetse fly facilities of Institut Pasteur and LSTM. At both colonies, infected tsetse fly PV were harvested between 21 and 28 days after infection (A and C), and parasite life cycle stages of cells from these infected PV were blindly quantified from three fixed and DAPI (4′,6-diamidino-2-phenylindole)-stained slides per cell line with approximately 100 cells per slide (B and D). (A) A total of 657 flies were dissected at Institut Pasteur (*n* = 3; error bars indicate standard deviations). (B) L262P/L262Pγ clone 1 was assessed, but no cells were detected in the PV after fixation due to the very low initial parasite density, and L262P/L262Pγ clone 2 was not assessed, as no infected PV were detected (see panel A). ND, none detected; MS, mesocyclic; DE, dividing epimastigote; E/LE, epimastigote/long epimastigote; SE, short epimastigote. Error bars indicate standard deviations. (C) One hundred fifty flies per repeat (50 per cell line) were dissected at LSTM.

**FIG 6:**
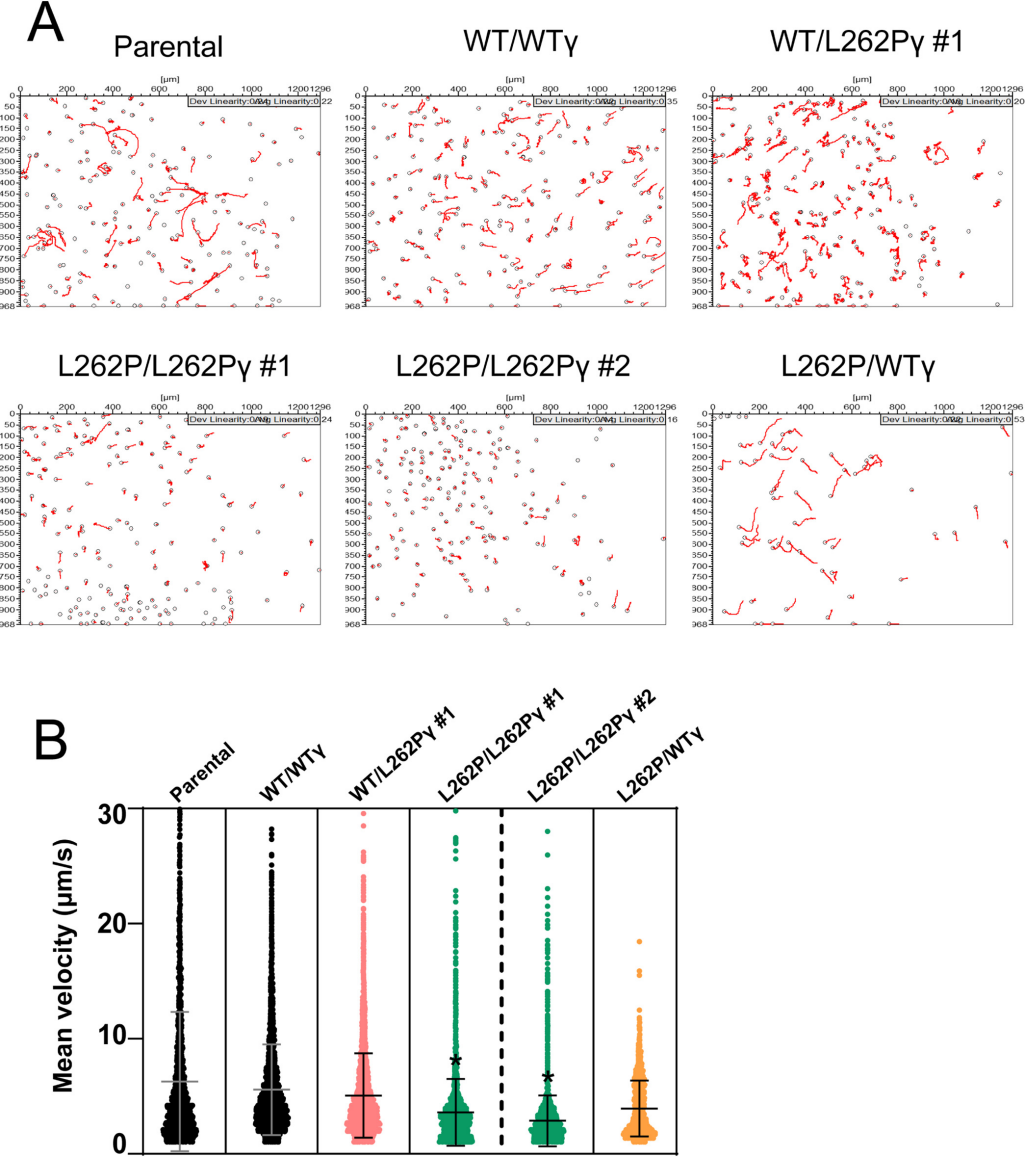
Motility analysis of parasites isolated from the fly midgut (*ex vivo*). (A) Representative tracks taken from videos of cells from tsetse fly midguts dissected at day 9 postinfection at Institut Pasteur. (B) Measurement of curvilinear velocity (VCL; calculated as mean velocity for each cell) from tracked videos of *ex vivo* PCF cells. *P* values were determined using ANOVA between cell lines. *, *P* < 0.05 (significant reduction in motility in comparison to the other cell lines). Error bars indicate standard deviations. At least 11 movies were taken per fly posterior midgut, with at least 3 flies dissected per strain. At least 1,546 cells were tracked per cell line.

We then assessed the ability of the heterozygous and homozygous L262Pγ mutants to colonize the PV and to continue their development. Whereas a high proportion of midgut infections manifested into PV infections with cells having at least one WTγ allele, infections with L262P/L262Pγ cells produced either very low numbers of (clone 1) or no (clone 2) infected PV (Fig. 5A). As predicted, adding back a WTγ allele to an L262P/L262Pγ cell line restored a normal PV infection rate. Infections performed at the other tsetse colony also produced substantially reduced PV infection rates for L262P/L262Pγ parasites that were also rescued in a WTγ add-back cell line (Fig. 5C). Tracks recorded from cells released from the midgut showed that L262P/L262Pγ cells displayed significantly less motility than other cell lines within the midgut, although it was notable that there was a large proportion of cells with relatively low motility for all cell lines (Fig. 6). Of note, rescue cell line L262P/WTγ showed a somewhat bimodal distribution, with an apparently distinct subpopulation of cells with higher motility. This could reflect heterogenous expression of the transgene in the parasite population. Overall, we conclude that the low PV infection rate is correlated with a decreased motility of these cells.

To assess whether capacity for OXPHOS also affected differentiation, we quantified the various parasite stages found in the PV. At the Institut Pasteur facility, we observed that cells with at least one WTγ allele could differentiate into long mesocyclic trypomastigote forms as well as into long and short epimastigote forms (Fig. 5B). WT/L262Pγ cells showed higher proportions of epimastigote stages than WT/WTγ cells; however, introduction of a single WTγ allele in the L262P/L262Pγ background (L262P/WTγ) resulted in a percentage of epimastigote cells that was more similar to that in the WT/WTγ cells. The life cycle stages of L262P/L262Pγ cells found in the PV could not be assessed for experiments at this fly colony due to the extremely low level of infections found in this organ. The PV infection rate for the L262P/L262Pγ mutant at the LSTM fly colony allowed us to assess the proportion of epimastigote forms for these cells and for parental and WTγ add-back cells as key controls. Interestingly, epimastigotes could be seen at approximately the same proportion in all these cell lines (Fig. 5D), despite the severely reduced levels of PV infection with L262P/L262Pγ cells. This suggests that, if able to migrate to the PV, L262P/L262Pγ cells can physically progress to the epimastigote stage of the life cycle. Thus, whether a reduced capacity of these cells for OXPHOS affects the efficiency of differentiation to the epimastigote stage remains uncertain from these data.

### Ability of L262Pγ F_1_F_o_-ATP synthase mutants to complete the life cycle.

**FIG 7:**
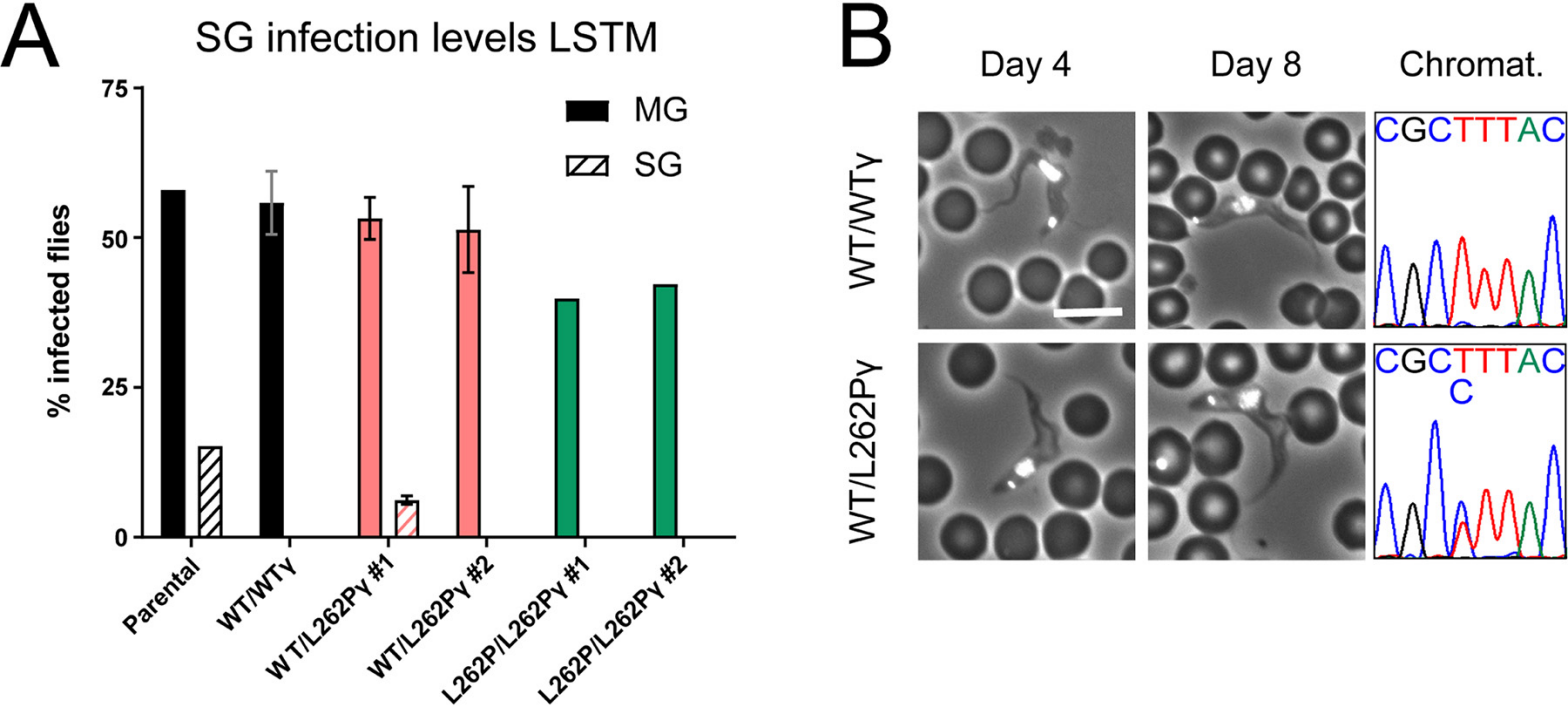
Assessment of fly salivary gland infectivity of parasites with ATP synthase mutations. (A) Infected tsetse fly midguts (MG) and salivary glands (SG) were harvested 4 weeks after infection. For infections with WT/WTγ, WT/L262Pγ clone 1, and WT/L262Pγ clone 2, *n* = 2. For the remaining infections, *n* = 1. Error bars indicate standard deviations. Around 200 flies were infected with one blood cryostock containing stumpy-form trypanosomes per cell line and replicate. Infections were performed at LSTM. (B) Mice were infected with metacyclic-form trypanosomes isolated from fly salivary glands from panel A. Tail snips were performed daily to assess parasitemia, and DAPI-stained blood smears were collected on days stated. Bar, 10 μm. The sequencing chromatograms on the right confirm the heterozygous ATP synthase subunit γ genotype (CTT = leucine; CCT = proline).

To investigate whether the life cycle could be completed by L262Pγ-expressing cell lines, we dissected infected tsetse flies at 28 days postinfection. Around 12% of tsetse flies fed with parental stumpy cells had detectable parasites in their salivary glands (Fig. 7A), confirming the ability of this parental cell line to complete the T. brucei life cycle. WT/L262Pγ clone 1 produced a lower salivary gland infection rate at an average of 6%. No infected salivary glands were found in flies that had been fed WT/WTγ, WT/L262Pγ clone 2, or L262P/L262Pγ parasites, despite these flies having established midgut infections. The lack of salivary gland infections for the WT/WTγ and one of the WT/L262Pγ cell lines suggests that the capacity for this step in the life cycle progression is perhaps unstable and prone to loss in experimental cell lines (although for a given cell line and experiment, salivary gland infection results are reproducible). As a consequence, the significance of the lack of infectivity of L262P/L262Pγ cells is uncertain.

To confirm that the metacyclic form trypanosomes found within the salivary glands in the parental strain and in WT/L262Pγ clone 1 were indeed animal transmissible, we harvested these forms and used them to infect mice. Slender BSF trypanosomes were visible in both tail blood smears from day 4, with stumpy forms being visible in both strains from day 8 (Fig. 7B). To ensure that cells of the original genotype WT/L262Pγ had not reverted to the WT/WTγ genotype, we confirmed by sequencing that both WT and L262Pγ alleles were detectable at the DNA level. Hence, T. brucei organisms with a WT/L262Pγ genotype can complete their parasitic life cycle.

To the best of our knowledge, this is the first published example of a comparison of infection results from two distinct tsetse fly colonies. However, not all cell lines were challenged each time due to fly number restraints and to the extremely laborious nature of the experimental tasks. In total, result trends observed in parallel with the same strains were similar between the tsetse fly colonies at LSTM, Liverpool, and the Institut Pasteur, Paris. They differed only in the degree to which the L262P/L262Pγ mutation affected midgut and PV infections. A decrease in midgut infection level was seen for the Institut Pasteur infections (Fig. 4B) compared to those performed at LSTM (Fig. 4A). This correlated with PV infection results, where the L262P/L262Pγ cells barely established a PV infection at Institut Pasteur (Fig. 5A), in contrast to LSTM, where more substantial PV infections were found, but at a lower level than those found in other cell types (Fig. 5C). These subtle differences could be explained by the different intrinsic vector competences of the two colonies and/or by differences in the infection and maintenance protocols (infection vehicle, blood meal type, and frequency).

## DISCUSSION

The kinetoplast (or kDNA) of the sleeping sickness parasite T. brucei encodes essential subunits of respiratory chain complexes, the F_1_F_o_ ATP synthase and the mitoribosome, which is a property it has in common with mitochondrial DNA of other eukaryotes. Although mitochondrial activity shows dramatic changes during the complex life cycle of the parasite, multiple *in vitro* studies have confirmed that the major proliferative stages in the mammalian bloodstream (long slender BSF) and in the tsetse fly vector midgut (PCF) depend on kDNA-encoded gene products to manufacture ΔΨm (BSF and PCF) and mitochondrial ATP (PCF). In the BSF, dependence on kDNA for viability can be overcome by compensatory changes in the nucleus-encoded F_1_ subunit γ. We utilized a panel of BSF mutants with various defects in kinetoplast-encoded functions (Table 1) combined with a number of multiple experimental challenges to address whether the compensatory mutation that functions in the BSF can compensate for kDNA loss in PCF and to what extent capacity for ATP production by OXPHOS is required for PCF differentiation and viability *in vivo*, as well as for cyclical development *in vivo* in the tsetse fly.

### Parasites heterozygous for the compensatory subunit γ mutation can complete the life cycle.

A cell line heterozygous for the mutation that enables viability of kDNA^0^ BSF parasites, WT/L262Pγ clone 1, was able to complete the life cycle within the fly and produce animal-infective metacyclic parasites. Growth, capacity for OXPHOS and motility *in vitro*, and midgut infection prevalence all were unimpaired by the WT/L262Pγ genotype. Although WT/L262Pγ parasites displayed a higher rate of epimastigote-form infection in the PV than WTγ parasites, this observation was probably not linked to the heterozygous genotype itself, as another heterozygous cell line, the WTγ add-back cell line (L262P/WTγ), showed levels of epimastigote forms that were more similar to those in the WTγ cell lines.

Approximately 25% of flies with parental strain midgut infections developed salivary gland infections (or ∼12.5% of the total number of flies fed with parasites), which is consistent with infection levels with this strain reported elsewhere (111). In flies infected with WT/L262Pγ clone 1 parasites, approximately 10% of flies with a midgut infection developed a salivary gland infection. However, as only the parental line and one WT/L262Pγ clone were able to differentiate into the metacyclic stage, and as the ability to invade the salivary glands seems to be a fragile trait that can be lost in laboratory-adapted cell lines, it cannot be ruled out that these differences in infection rate could be due to clone-specific differences unrelated to the genetic manipulation. Nonetheless, our results clearly demonstrate that parasites with heterozygous L262Pγ mutations enabling kDNA independence can be transmitted by tsetse flies. T. brucei parasites with this or other mutations that obviate kDNA in BSFs are much less sensitive to classes of drugs used in the field to treat animal disease, including isometamidium and homidium, since these drugs, at least in part, target parasite kDNA (112). Exposure to drugs that target kDNA, however, would cause loss of kDNA from WT/L262Pγ cells, thus preventing transmission by tsetse flies, as confirmed in the present study (see below). Hence, although a drug-resistant phenotype caused by a heterozygous subunit γ genotype could initially be spread by cyclical transmission, once in a host treated with these drugs, parasites would continue to proliferate, but the opportunity for further tsetse transmission from that host would stop. If such mutations were selected for in an animal-infectious strain of Trypanosoma brucei *brucei*, there would also be scope for sexually recombining with human-infective parasites to produce parasites with increased drug resistance able to infect humans (113, 114).

### Requirement for F_1_F_o_-ATP synthase complexes through the life cycle.

A comparison of the phenotypes observed for cells with a homozygous L262P F_1_ subunit γ mutation versus null mutants for F_o_ subunit Tb1 provided insight into the requirement for F_1_F_o_ complexes for life cycle completion. First, L262P/L262Pγ cells (unlike WT/WTγ and WT/L262Pγ cells) grow poorly under glucose-depleted conditions, which increase dependence on OXPHOS (28, 37). Tb1^−^/Tb1^−^ cells, on the other hand, die rapidly under these conditions. Second, early in differentiation to PCF *in vitro*, L262P/L262Pγ cells maintain higher cellular ATP concentrations than Tb1^−^/Tb1^−^ cells. Third, L262P/L262Pγ cells sustain midgut populations in the glucose-depleted tsetse fly midgut, although these are less dense, and cells are less motile by day 9 than in flies infected with WT/WTγ or WT/L262Pγ cell lines. Tb1^−^/Tb1^−^ cells, in contrast, were unable to establish or sustain midgut infections. Together, these results demonstrate that, first, differentiation to PCF and tsetse infectivity depends on functional F_1_F_o_ ATP synthase complexes, which is consistent with the observation that stumpy parasites treated with the F_1_F_o_ inhibitor oligomycin were unable to reenter the cell cycle during differentiation to PCF cells (68). Second, the L262Pγ mutation causes a less profound defect in F_1_F_o_-ATP synthase function than the loss of Tb1. This suggests that, whereas the F_1_F_o_-ATP synthase is nearly completely disrupted in the absence of Tb1 (107, 109), the F_1_F_o_ complex retains some functionality in L262P/L262Pγ cells, as these cells can still generate enough ATP mitochondrially to survive under these conditions.

In principle, the effect of the mutations on mitochondrial ATP production could be direct (via F_1_F_o_-ATP synthase participation in OXPHOS), indirect (via an impact on substrate level phosphorylation), or a combination of both. Crude mitochondrial extracts from L262P/L262Pγ cells barely produce ATP via OXPHOS, with ATP production levels close to those in the presence of an ATP synthase inhibitor. This is consistent with the expected partial uncoupling between F_1_ and F_o_ that is caused by the disrupted interaction between the mutated γ subunit and F_1_ headpiece (84–87). On the other hand, like stumpy cells (24, 115, 116), newly differentiated L262P/L262Pγ PCF cells are able to metabolize α-ketoglutarate via mitochondrial substrate level phosphorylation to generate ATP. Indeed, we found that L262P/L262Pγ clone 2 cells produce ATP from α-ketoglutarate to an extent similar to that of WTγ cells. As L262P/L262Pγ cells can establish infection in the tsetse midgut and progress to the PV, we postulate that the capacity for substrate-level phosphorylation in the absence of glucose may be higher *in vivo* than *in vitro*; threonine or glutamine concentrations, for example, may be higher per trypanosome in the midgut (117, 118). The presence of symbiotic bacteria in the tsetse fly midgut and the influence of fly digestion may also impact the metabolism of midgut-associated parasites (35, 119, 120). Nutrients may also be depleted faster within liquid culture than in the fly midgut.

Why would L262P/L262Pγ cells and Tb1^−^/Tb1^−^ cells differ in their capacity for mitochondrial ATP production via substrate-level phosphorylation? In L262P/L262Pγ PCF cells, ΔΨm can be generated by electron transport chain components, namely, respiratory complexes III and IV (44, 45). In these cells, the partial uncoupling between the F_1_ and F_o_ parts would be expected to generate a proton leak through F_o_ into the matrix (84–87). However, the viability of these PCF cells and the fact that they can produce ATP in the presence of α-ketoglutarate suggest that a ΔΨm of sufficient magnitude is maintained to support metabolite transport and mitochondrial protein import for substrate-level phosphorylation. In Tb1^−^/Tb1^−^ cells, however, the nearly complete loss of F_o_ from ATP synthase complexes (107, 109) might cause sustained hyperpolarization of the inner mitochondrial membrane, as the ΔΨm generated by complex III and IV cannot be dissipated by proton movement through the F_o_ moiety (121–123). Hyperpolarization would interfere with metabolite exchange and mitochondrial protein import and cause reactive oxygen species (ROS) generation and enhanced oxidative stress, thus damaging cells in a multitude of ways (124). Indeed, persistent hyperpolarization is an acknowledged cell fate and death checkpoint (125–128). In addition, the profound inability of Tb1^−^/Tb1^−^ cells to survive as PCF *in vitro* or *in vivo* may be influenced by the substantial remodeling of the mitochondrion required during BSF-to-PCF differentiation (9). During this differentiation, the tubular BSF mitochondrion expands in volume and becomes elaborated into a branched network structure with abundant cristae (15, 115, 129), which are important for efficient OXPHOS (130–132). Dimerization of the trypanosome F_1_F_o_ complex may be critical for folding of the inner mitochondrial membrane and cristae formation and may critically involve F_o_, as in other organisms (133–138). The absence of Tb1 and, consequently, F_o_ in these cells could interfere with the remodeling of the inner mitochondrial membrane during differentiation, preventing the ordered processes required from being completed. The mutants with progressive developmental defects generated here therefore will be a useful resource for future studies aimed at correlating kDNA-encoded functions with the changes in mitochondrial physiology and ultrastructure that are a hallmark of trypanosome differentiation.

Although the reduced ATP production levels that we observed for L262P/L262Pγ cells are sufficient to permit colonization of the midgut, they are correlated with a progressive motility defect that appears to decrease the migration efficiency of the cells in the fly. Only a low level of PV infection was detected in flies infected with L262P/L262Pγ cells. Swimming ability has been shown to be dictated by the availability of ATP for the dynein motor in numerous eukaryotic flagellate organisms (139–143). In T. brucei, a dynein motor mutant has a motility defect in culture and consequently is unable to migrate to the foregut and PV or infect the salivary glands (144). Furthermore, efficient ATP generation is required for motility of T. brucei (145). The ability to efficiently metabolize proline and alternative carbon sources may be particularly important as the parasite tries to migrate to compartments in the tsetse fly that could be less nutrient rich than the midgut, especially at the end of the digestive process (34, 35). The lower infection level found in the L262P/L262Pγ-infected midguts may also play a role in reduced migration as per the bottleneck model of T. brucei differentiation and migration in the tsetse fly (55). In our *ex vivo* motility analysis, we noticed a pronounced heterogeneity among parasite populations for all cell lines, including the parental line, which could be a contributing factor to the bottleneck effect.

In addition to motility, substantial amounts of ATP are also required for cell cycle progression and differentiation (146). DNA replication complexes and kinesin proteins governing the vast morphological and organelle rearrangements depend on translocations fueled by ATP (147–150). Indeed, the progressive cellular ATP decline over time seen in L262P/L262Pγ cells is probably due to the large ATP expenditure necessary for motility and cell cycle progression, and the inefficiency of ATP generation in this cell line. *In vivo*, L262P/L262Pγ cells can differentiate into epimastigotes in a limited fashion, agreeing with recent evidence that OXPHOS may not be essential for this life cycle stage (35). The presence of L262P/L262Pγ epimastigotes was unexpected considering that developmental progression in the insect may be driven by ROS production (63) and that the presence of a L262Pγ-induced proton leak would be hypothesized to neutralize ROS generation (151).

L262P/L262Pγ cell lines could not infect tsetse salivary glands despite these cell lines being able to sustain a midgut infection for over 4 weeks in these flies. The significance of this observation is uncertain, as it is common to observe a loss of fly infectivity in cultured T. brucei strains (152), as observed here for WT/WTγ and WT/L262Pγ clone 2 cell lines. As all cells had been maintained previously in BSF culture, it is possible that there was selection for parasites that had lost genes required for progression from the PCF in the tsetse midgut.

### The requirement for kDNA.

Stumpy kDNA^0^ cells were consistently unable to (i) establish a midgut infection and (ii) differentiate into viable PCF cells *in vitro* after induction with CA, dying within 24 h. The kDNA^0^ stumpy cells express PAD1 (24) and do have a functional signaling pathway for differentiation, as they respond to CA by expressing EP procyclin at a time point comparable to that seen with kDNA^+^ cell lines, as reported here and elsewhere (99, 102). However, kDNA^0^ parasites do not effectively reenter the cell cycle after differentiation from the cell cycle-arrested stumpy BSF. EP procyclin expression appears to be independent of kDNA, but effective reentry into the cell cycle seems to require either the presence of the kinetoplast structure or a kDNA-encoded function, which is in agreement with the work of Timms et al. (68). We suggest that this is due to a complete failure to generate a ΔΨm. In the presence of functional kDNA, both the slender and stumpy BSFs generate ΔΨm by operating the F_1_F_o_-ATP synthase in the reverse direction (17–19, 24, 153). The enzyme switches to the forward direction during stumpy to PCF differentiation (24, 154), and ΔΨm is then generated in a canonical fashion by respiratory complexes III and IV. According to the current model, the kDNA^0^ long slender BSF can maintain ΔΨm independently of F_o_ via the electrogenic action of AAC-mediated ATP^4−^/ADP^3−^ exchange across the inner mitochondrial membrane when supported by efficient ATP hydrolysis via an F_1_ enzyme bearing a compensatory mutation in subunit γ (17, 24, 66, 82, 83). However, ΔΨm is not generated in kDNA^0^ stumpy cells, probably due to mitochondrial substrate-level ATP production using α-ketoglutarate preventing the electrogenic import of cytosolic ATP by the AAC (24). In the absence of kDNA-encoded complex III and IV, kDNA^0^ PCF would be unable to generate ΔΨm for the same reason. We can conclude that all naturally occurring kDNA^−^ or kDNA^0^ variants of T. brucei, such as *T. b. evansi* and *T. b. equiperdum*, are intrinsically unable to undergo cyclical development in the tsetse fly.

## MATERIALS AND METHODS

### Cell line generation.

All cell lines used in this study were derived from culture-adapted pleomorphic T. brucei EATRO 1125 AnTat1.1 90:13 BSF parasites (Table S1) (99). Heterozygous cell lines with one F_1_F_o_-ATPase subunit γ allele (systematic TriTrypDB ID Tb927.10.180) with the L262P mutation (L262Pγ) and akinetoplastic (AK) versions of these cell lines were generated as detailed in reference 24. Homozygous L262Pγ cell lines were generated by transfecting WT/L262Pγ T. brucei with plasmid pEnT6-γL262P-BSD (blasticidin resistance marker) to replace the remaining endogenous WTγ allele with an L262Pγ copy. The WTγ add-back cell line was generated by releasing L262P/L262Pγ T. brucei cells from either puromycin or blasticidin selection before the transfection and subsequently transfecting them with plasmid pEnT6-γWT-PHL (phleomycin resistance marker). Both of these plasmids are based on the pEnT6 backbone (155) and contain either a L262Pγ gene or a wild-type version (WTγ), facilitating the replacement of an ATPase γ subunit allele. The replaced gene is expressed by read-through transcription of the endogenous locus and contains its native 5′ untranslated region (UTR) but the aldolase 3′ UTR.

To generate a Tb1 (Tb927.10.520)-null (Tb1^−^/Tb1^−^) cell line, pUC57-based plasmids containing either the blasticidin or the phleomycin resistance cassettes were designed and ordered from Biomatik (Fig. S2). Actin 5′ and 3′ UTRs flank the resistance cassettes, with 350 bp of the Tb1 5′ UTR and intergenic region (IGR) positioned upstream of this and 350 bp of the Tb1 3′ UTR and IGR positioned downstream of this. The first allele knockout (KO) construct was designed to target sequences distal to those employed by the second allele KO construct (156). These KO constructs were positioned inside HindIII restriction sites to enable digestion before transfection.

For the transfection, the Amaxa Nucleofector II (Lonza) was used with nucleofection solution (90 mM NaH_2_PO_4_, 5 mM KCl, 0.15 M CaCl_2_, 50 mM HEPES [pH 7.3]) and program Z-001. T. brucei EATRO 1125 AnTat1.1 90:13 BSF clones were selected after 4 days at 37°C under drug selection as necessary (2.5 μg/mL G418, 5 μg/mL hygromycin, 0.1 μg/mL puromycin, 5 μg/mL blasticidin, 5 μg/mL phleomycin) in HMI-9 medium (157) containing 10% (vol/vol) fetal calf serum (FCS; Gibco). ATPase γ genotypes were verified as detailed by Dewar et al. (24). Tb1 KO cell lines were verified by PCR and Western blotting.

### *In vitro* differentiation and cell culture.

Stumpy forms were generated in mice and were either used to make cryostocks containing a parasite population with approximately 90% stumpy bloodstream forms or purified from blood (24), as required. *In vitro* differentiation was performed by adding 6 mM *cis*-aconitate (CA) to each culture, and cultures were left at 27°C for 24 h. If samples contained blood cells, the flasks were balanced on one corner to allow blood cells to settle out of the medium. Newly differentiated PCF cells were washed and resuspended either in SDM79 containing 10 mM glucose (Invitrogen) (158) or in SDM80 at a density of at least 2 × 10^6^ cells per mL medium. SDM80 was made from SDM79 CGGGPPTA powder (GE Healthcare), an SDM79-based powder that lacks major carbon-containing components (sodium bicarbonate, glucose, glutamine, glutamate, proline, pyruvate, threonine, and sodium acetate). All carbon sources except glucose were added back into the solution at the following concentrations: pyruvate, 100 mg/L; l-proline, 615 mg/L; l-threonine, 394 mg/L; l-glutamine, 320 mg/L; l-glutamate, 24 mg/L; NaHCO_3_ 2 g/L; sodium acetate, 10 mg/L. The medium was supplemented with 50 mM *N*-acetyl d-glucosamine (Sigma) to prevent residual glucose uptake from FCS (159) and maintained under drug selection as necessary (15 μg/mL G418, 25 μg/mL hygromycin, 2 μg/mL puromycin, 10 μg/mL blasticidin, 2.5 μg/mL phleomycin). SDM79 and SDM80 were supplemented with 7.5 mg/L hemin and 10% FCS (Invitrogen), and cells were grown at 27°C continuously in these media, maintaining density over 2 × 10^6^/mL. For time courses, day 0 (d0) was defined as being after 24 h in HMI-9 at 27°C plus CA and then 24 h in SDM80 at 27°C.

### Protein expression analysis by flow cytometry and Western blotting.

For a quantitative measurement of EP expression, 2 × 10^6^ cells were harvested from +CA and −CA cultures at 0 h, 1 h, 2 h, 3 h, 6 h, 16 h, and 24 h after CA addition. Upon collection, samples were transferred to 5-mL polystyrene round-bottom tubes (BD Flacon 352052), centrifuged at 2,000 × *g* for 5 min, and washed in phosphate-buffered saline (PBS; pH 7.4; 137 mM NaCl, 2.7 mM KCl, 10 mM Na_2_HPO_4_, 1.8 mM KH_2_PO_4_). The cell pellet was fixed in 500 μL PBS with 2% formaldehyde and 0.05% (wt/vol) glutaraldehyde overnight. Cells were pelleted, washed three times in PBS, and blocked in PBS with 2% (wt/vol) bovine serum albumin (BSA) for 1 h. After a PBS wash, cells were stained with PBS containing 2% BSA and 1/500 anti-EP procyclin monoclonal antibody (Cedarlane Laboratories) for 1 h. Cells were washed in PBS, and then PBS containing 2% BSA and 1/1,000 anti-mouse IgG–Alexa 488 secondary antibody was added and left for 1 h. Cells were then washed and resuspended in 500 μL PBS containing 5 μg/mL Hoechst DNA staining dye (Life Technologies). After a 30-min incubation at room temperature, cells were analyzed by flow cytometry at an excitation wavelength (λ_ex_) of 495 nm and an emission wavelength (λ_em_) of 519 nm using a Becton Dickinson LSR II machine with BD FACSDiva software, and 2 × 10^4^ events per sample were measured. Results were analyzed with FlowJo software (BD Biosciences).

Western blotting was performed as per Dewar et al. (24). Antibodies used were anti-EP procyclin (1:500), anti-ATP synthase Tb1 (1:2,000) (160), anti-ATP synthase Tb2 (1:2,000) (160), anti-ATP synthase β (1:2,000) (160), anti-COXIV (1:1,000) (41), anti-COXVI (1:500) (44), anti-EF1α (1:7,000) (Millipore), and anti-mtHSP70 (1:2,000) (161).

### Tsetse fly handling and infections.

To make the infective parasite stabilates, mouse blood containing stumpy-form trypanosomes was harvested, mixed with 2% (wt/vol) sodium citrate to act as an anticoagulant, and mixed 1:1 with HMI-9 containing 30% (vol/vol) FCS and 7.5% (vol/vol) glycerol for freezing at −80°C. Blood meals for tsetse fly infections were prepared as follows: one 200-μL aliquot per cell line was thawed at room temperature and mixed with ∼5 mL room-temperature, sterile, defibrinated horse blood at the LSTM or with ∼2 mL room temperature heat-inactivated fetal calf serum at the Institut Pasteur.

At the LSTM, the Glossina morsitans morsitans (Westwood; origin, Kenya) colony was maintained at 26 ± 2°C and 68 to 78% relative humidity, with a 12-h light/12-h dark cycle, and was fed triweekly on sterile, defibrinated horse blood in the dark using a sterile silicon membrane and a heated mat set to 37°C. All flies used in this study were newly emerged, teneral (unfed) male adults. Each experimental group was fed one infected blood meal containing predominantly stumpy-form parasites when flies were at 0 to 24 h after emergence. To obtain midgut infections, flies were offered a parasite-infected blood meal for 10 min, and then 24 h after feeding, flies were chilled to 4°C to remove unfed flies (identified by a nonscarlet abdomen). Flies with a visible blood meal were maintained at 27°C until dissection on day 9 postinfection and fed uninfected blood meals as described above every 2 to 3 days. For salivary gland infections, flies were allowed to feed on an infected blood meal for 15 min. Flies were chilled to 4°C at 24 h after the blood meal to remove unfed flies. Flies were maintained as described above and dissected 4 weeks postinfection.

At the Institut Pasteur, *G. m. morsitans* tsetse flies (Westwood; origins, Zimbabwe and Burkina Faso) were maintained in Roubaud cages at 27 ± 1°C and 70% ± 5% relative humidity, with a 12-h light/12-h dark cycle, and fed twice a week through a silicone membrane with sterile, mechanically defibrinated sheep blood at 37°C. Teneral males (between 24 h and 72 h postemergence) were allowed to ingest BSF parasites in heat-inactivated fetal calf serum at 37°C through a silicone membrane for 10 min during their first meal, and unfed flies were removed after feeding. When possible, flies were starved for at least 24 h before being dissected. For dissection, midguts and PVs were rapidly isolated and placed in distinct drops of PBS with 0.1% glucose as previously described (54, 61). Isolated tissues were assessed by microscopy (40×) and imaged or allowed to infuse for 5 min in a wet chamber before removal and collection of the remaining PBS drop containing trypanosomes for video recording (tracking analyses; see below) (162).

To infect mice with metacyclic parasites harvested from tsetse flies, infected salivary glands were collected and pooled in ice-cold SDM79. Frozen stocks of these salivarian parasites were prepared by adding glycerol to a final concentration of 7.5% (vol/vol) before freezing at −80°C. Mouse infections, blood smears, and harvesting were performed as detailed in reference 24.

### Motility tracking and analysis (*in vitro* and *ex vivo*).

*In silico* 2D tracking was performed as previously described (144). Briefly, PCF trypanosomes were sampled from cultures or freshly isolated from tsetse fly tissues, suspended on noncoated glass slides in a drop of PBS with 0.1% (wt/vol) glucose, and maintained at 27°C. Parasites were observed under the 10× objective of an inverted DMI-4000B microscope (Leica) coupled to an ORCA-03G (Hamamatsu) or a PRIM95B (Photometrics) camera. Video recording was performed within 15 min after isolation, and the focal plane was set up in the middle of the PBS drop containing parasites in order to prevent the recording of attached cells at the base of the drop or drifting cells at the top of the drop. For each cell line, 10 to 20 movies were recorded for 20 s (50 ms of exposure). Movies were converted with the MPEG Streamclip V.1.9b3 software (Squared 5) and analyzed with the medeaLAB CASA Tracking V.5.5 software (medea AV GmbH) for quantifying the mean velocity of 200 individual trypanosomes per movie over 149 successive frames. Statistical analyses and plots were performed with XLSTAT 2019.2.01 (Addinsoft) in Excel 2016 (Microsoft) or Prism V8.2.1 (GraphPad). Statistical analyses include two-sided analyses of variance (ANOVA) with Tukey’s *ad hoc* posttests for intergroup comparison at 95% confidence.

### *In organello* ATP production and whole-cell ATP quantification assays.

The ATP quantification assay was performed as per the protocol for the CellTiter-Glo 3D cell viability assay kit (Promega), using 1 × 10^4^ cells per sample. *In organello* ATP production assays were performed using the CLSII ATP bioluminescence assay kit (Roche) and digitonin extractions of 1 × 10^7^ cells per sample (110). Statistical analyses involved one-sided ANOVA with Tukey’s *ad hoc* posttests for intergroup comparison at 95% confidence.
