# Supplementary material for: Oxidative Phosphorylation Is Required for Powering Motility and Development of the Sleeping Sickness Parasite Trypanosoma brucei in the Tsetse Fly Vector
Source: mBio. 2022 Jan 11;13(1):e02357-21. doi: 10.1128/mbio.02357-21 (PMC8749461; doi:10.1128/mbio.02357-21)
Supplement: TABLE S1 [file mbio.02357-21-st001.pdf]

Table S1. Cell lines used in this study.

Cell line	Derived from:	Genotype	Description
Parental	-	-	EATRO 1125AnTat 1.1 90:13
WT/WTγ	EATRO 1125AnTat 1.1 90:13	ATPγ/Δatpγ::atpγWT +PURO	One F1FO-ATP synthase subunit γ allele replaced with a WT copy and the puromycin resistance gene (PURO)
WT/L262Pγ #1	EATRO 1125AnTat 1.1 90:13	ATPγ/Δatpγ::atpγL262P +PURO	One F1FO-ATP synthase subunit γ allele replaced with a copy with the L262P mutation and the puromycin resistance gene (PURO)
WT/L262Pγ #2	EATRO 1125AnTat 1.1 90:13	ATPγ/Δatpγ::atpγL262P +PURO	One F1FO-ATP synthase subunit γ allele replaced with a copy with the L262P mutation and the puromycin resistance gene (PURO)
L262P/L262Pγ #1	WT/L262P #3	Δatpγ::atpγL262P +BSD/Δatpγ::atpγL262P +PURO	One F1FO-ATP synthase subunit γ allele replaced with a copy with the L262P mutation and the puromycin resistance gene (BSD)
L262P/L262Pγ #2	WT/L262P #3	Δatpγ::atpγL262P +BSD/Δatpγ::atpγL262P +PURO	One F1FO-ATP synthase subunit γ allele replaced with a copy with the L262P mutation and the puromycin resistance gene (BSD)
L262P/WTγ	L262P/L262P #2	Δatpγ::atpγL262P +BSD/Δatpγl262p::atpγWT +PHL	One F1FO-ATP synthase subunit L262Pγ allele replaced with a WT copy with the puromycin resistance gene (PHL)
WT/L262PγkDNA0 #1	WT/L262P #1	ATPγ/Δatpγ::atpγL262P +PURO	Cell line WT/L262Pγ #1 induced to lose kDNA through exposure to acriflavine
WT/L262PγkDNA0 #2	WT/L262P #2	ATPγ/Δatpγ::atpγL262P +PURO	Cell line WT/L262Pγ #2 induced to lose kDNA through exposure to acriflavine
Tb1-/Tb1- #1	WT/L262P #1	ATPγ/Δatpγ::atpγL262P +PURO<br>Δtb1::BSD/Δtb1::PHL	Cell line WT/L262Pγ #1 with BSD and PHL resistance genes present in Tb1 loci
Tb1-/Tb1- #2	WT/L262P #1	ATPγ/Δatpγ::atpγL262P +PURO<br>Δtb1::BSD/Δtb1::PHL	Cell line WT/L262Pγ #1 with BSD and PHL resistance genes present in Tb1 loci
Tb1-/Tb1- #3	WT/L262P #1	ATPγ/Δatpγ::atpγL262P +PURO<br>Δtb1::BSD/Δtb1::PHL	Cell line WT/L262Pγ #1 with BSD and PHL resistance genes present in Tb1 loci
