# Supplementary material for: Oxidative Phosphorylation Is Required for Powering Motility and Development of the Sleeping Sickness Parasite Trypanosoma brucei in the Tsetse Fly Vector
Source: mBio. 2022 Jan 11;13(1):e02357-21. doi: 10.1128/mbio.02357-21 (PMC8749461; doi:10.1128/mbio.02357-21)
Supplement: FIG S1 [file mbio.02357-21-sf001.pdf]

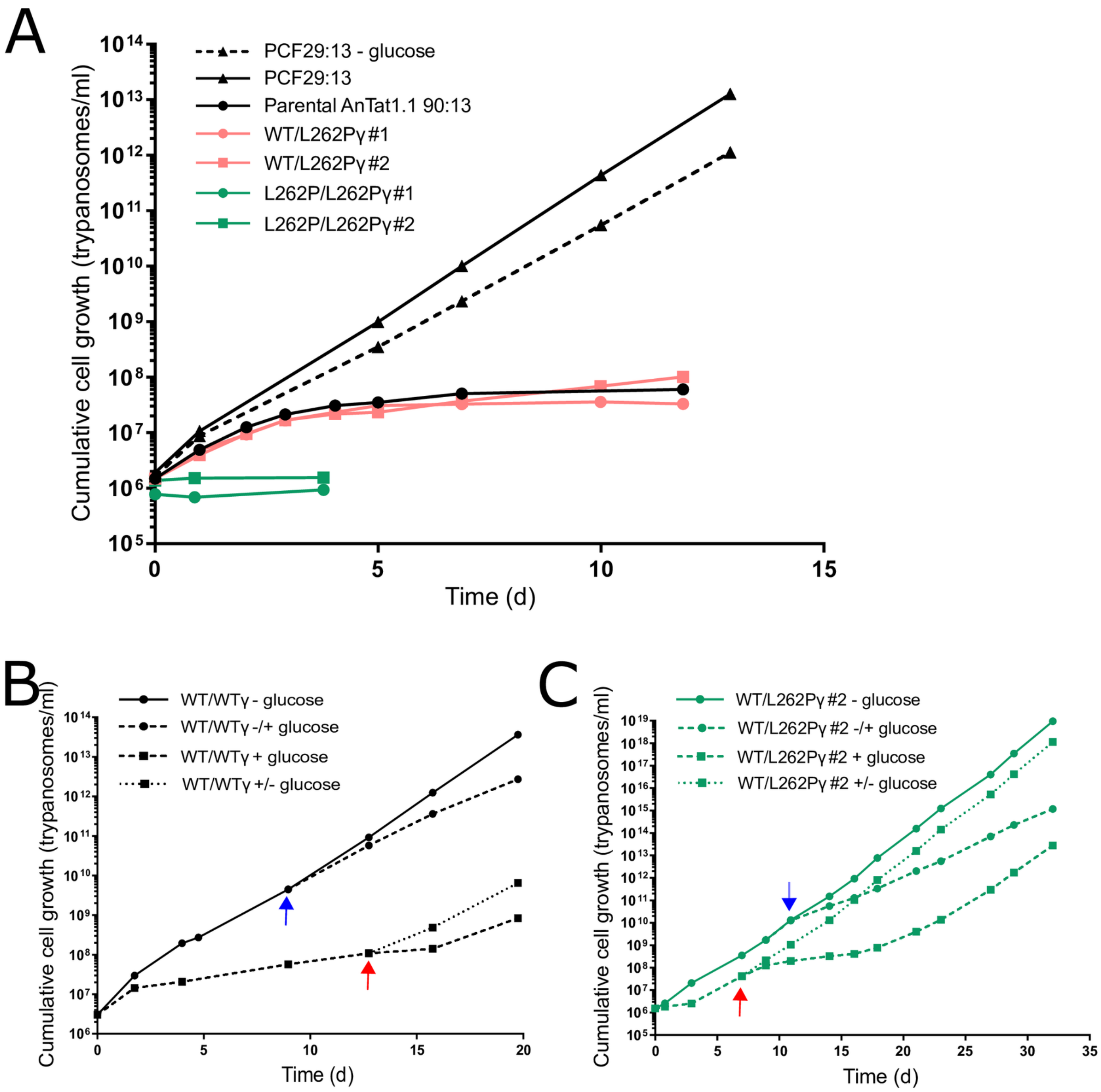

Fig S1. Effect of glucose on growth rates of freshly differentiated PCF cells. (A) Cells were treated with 6 mM CA in HMI-9 for 24 h and then transferred to SDM80 medium supplemented with 10 mM glucose (solid lines) at a density of $2x10^6$/ml (day 0). An established PCF T. brucei cell line, 29:13, was used as control that grows in either SDM80 (residual glucose, dashed lines) or SDM80 supplemented with glucose. Over the next 12-13 days, cells were counted once a day, and afterwards were diluted to a density of at least $3x10^6$/ml. (B, C) After induction of differentiation with CA, WT/WTγ (B) and WT/L262Pγ clone #2 parasites (C) were initially grown in either SDM80 (- glucose) or SDM80 supplemented with 10 mM glucose (+ glucose). Red arrows depict transfer of cells from SDM80 with glucose into SDM80 minus glucose (+/- glucose), and blue arrows depict transfer of cells into SDM80 minus glucose into SDM80 with glucose (-/+ glucose).
