# Supplementary material for: Oxidative Phosphorylation Is Required for Powering Motility and Development of the Sleeping Sickness Parasite Trypanosoma brucei in the Tsetse Fly Vector
Source: mBio. 2022 Jan 11;13(1):e02357-21. doi: 10.1128/mbio.02357-21 (PMC8749461; doi:10.1128/mbio.02357-21)
Supplement: FIG S2 [file mbio.02357-21-sf002.pdf]

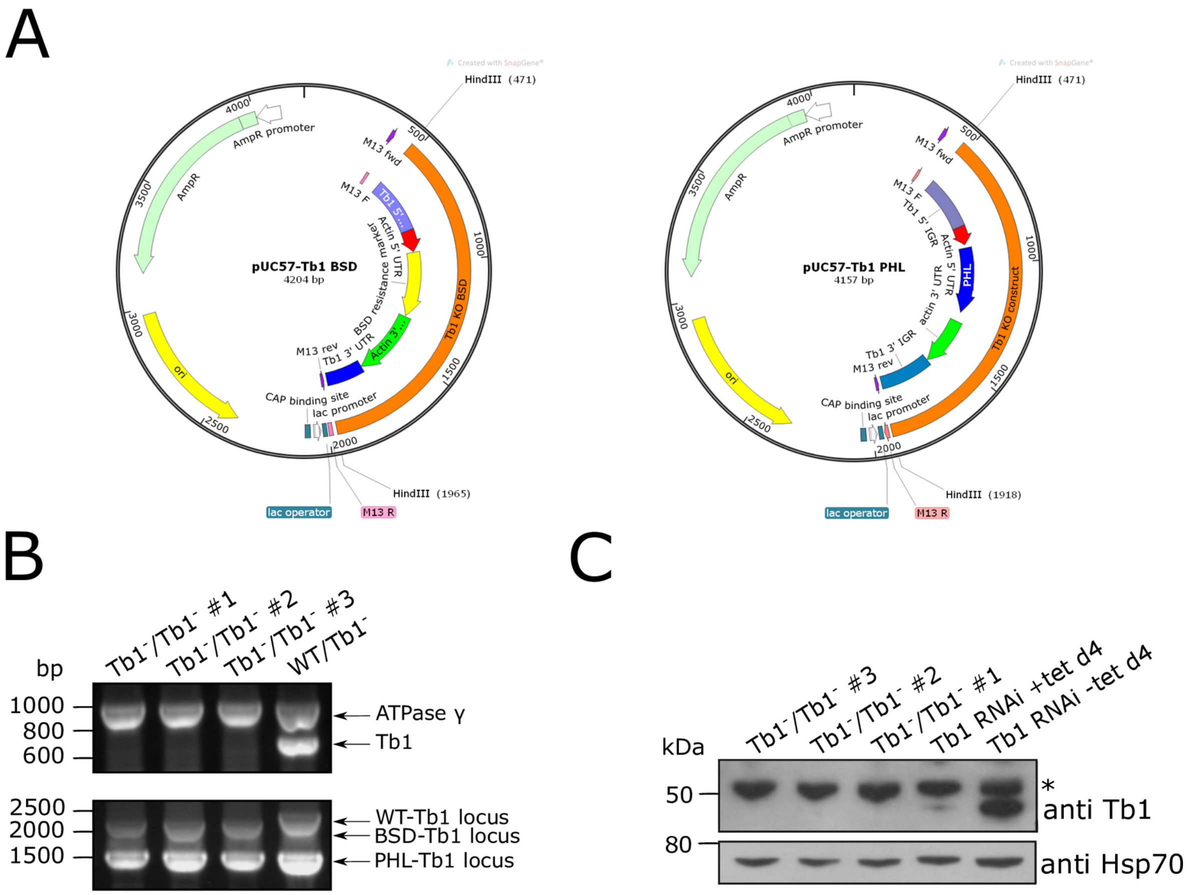

Fig S2. Generation and verification of Tb1 null mutants. (A) Tb1 knock-out plasmids produced by DNA synthesis (Biomatik). Drug selection markers (BSD, blasticidin resistance; PHL, phleomycin resistance) are flanked by actin 5' and 3' untranslated regions (UTR) for mRNA splicing and polyadenylation, respectively, and by Tb1 5' and 3' UTRs or intergenic regions (IGR) for homologous recombination. (B) PCR verification of three $Tb1^-/Tb1^-$ clones (#1, #2, #3). The top panel shows a duplex PCR with simultaneous amplification of a ~700-bp fragment of the Tb1 coding sequence and a ~900-bp fragment of the ATPase γ subunit gene as internal control. The bottom panel shows amplification of the Tb1 locus using primers flanking the 5' and 3' recombination sites. The wild type locus and the locus after replacement of the Tb1 coding sequence with the BSD and PHL genes give amplicons of 2400 bp, 2100 bp and 1540 bp, respectively. Genomic DNA from a $WT/Tb1^-$ single knock-out cell line is included as a control. (C) Western blot verification of three $Tb1^-/Tb1^-$ clones, using a Tb1 antibody and an Hsp70 antibody as control. Whole cell lysates of $2x10^6$ cells were analysed per lane. The asterisk indicates non-specific detection of a ~55-kDa protein by the Tb1 antibody. Lysates of an inducible Tb1 RNAi cell line (Hierro-Yap et al., 2021), uninduced and induced for 4 days for Tb1 ablation with tetracycline (tet), are shown as a control.
