# Supplementary material for: Oxidative Phosphorylation Is Required for Powering Motility and Development of the Sleeping Sickness Parasite Trypanosoma brucei in the Tsetse Fly Vector
Source: mBio. 2022 Jan 11;13(1):e02357-21. doi: 10.1128/mbio.02357-21 (PMC8749461; doi:10.1128/mbio.02357-21)
Supplement: FIG S3 [file mbio.02357-21-sf003.pdf]

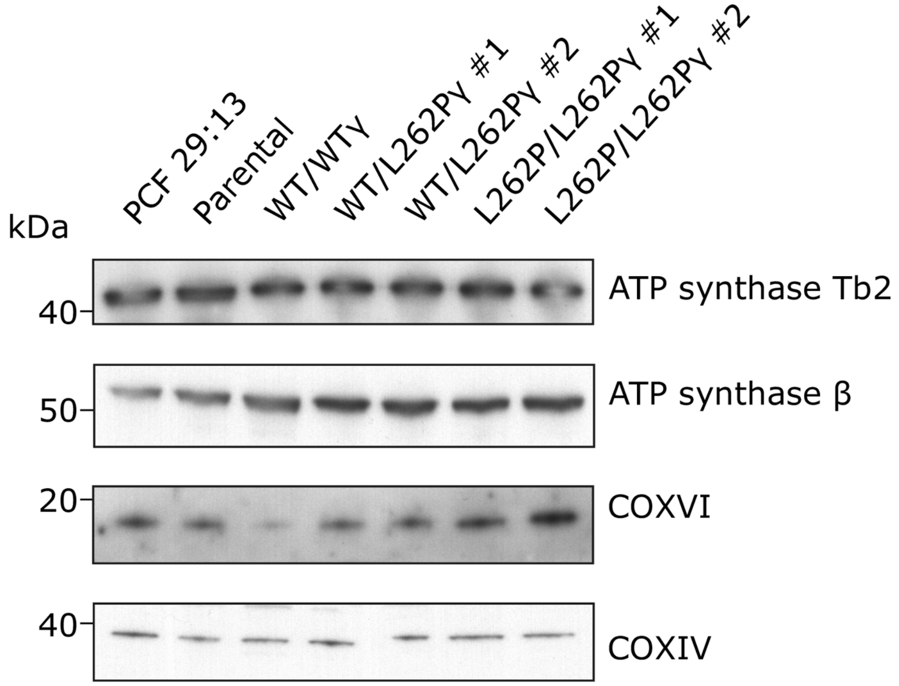

Fig S3. Abundance of F_1F_O-ATP synthase in γ subunit mutants. Cellular levels of mitochondrial respiratory complexes F_1F_O-ATP synthase and cytochrome oxidase (COX) assessed by probing a western blot with specific antibodies. Tb2 and β are subunits of the F_O and F_1 moieties of the ATP synthase, respectively. COX is represented by subunits IV and VI. Cells were harvested after 48 h in SDM80 medium and whole cell lysates of $2x10^6$ cells were loaded per lane.
