# Supplementary material for: Oxidative Phosphorylation Is Required for Powering Motility and Development of the Sleeping Sickness Parasite Trypanosoma brucei in the Tsetse Fly Vector
Source: mBio. 2022 Jan 11;13(1):e02357-21. doi: 10.1128/mbio.02357-21 (PMC8749461; doi:10.1128/mbio.02357-21)
Supplement: TABLE S2 [file mbio.02357-21-st002.pdf]

**Table S2. Statistical analysis by one-way ANOVA of selected data from Fig 2A.**

**Succinate**

| | WT/WTγ | WT/L262Pγ #2 | L262P/L262Pγ #1 | L262P/L262Pγ #2 | L262P/WTγ |
|---|---|---|---|---|---|
| **WT/WTγ** | | 0.0072 | **<0.0001** | **<0.0001** | 0.2216 |
| **WT/L262Pγ #2** | | | <0.0001 | <0.0001 | 0.0001 |
| **L262P/L262Pγ #1** | | | | 0.3673 | **<0.0001** |
| **L262P/L262Pγ #2** | | | | | **<0.0001** |

**Succinate + pyruvate**

| | WT/WTγ | WT/L262Pγ #2 | L262P/L262Pγ #1 | L262P/L262Pγ #2 | L262P/WTγ |
|---|---|---|---|---|---|
| **WT/WTγ** | | 0.0005 | **<0.0001** | **0.0429** | 0.0127 |
| **WT/L262Pγ #2** | | | <0.0001 | <0.0001 | 0.6589 |
| **L262P/L262Pγ #1** | | | | 0.1465 | **<0.0001** |
| **L262P/L262Pγ #2** | | | | | **0.0001** |

**Notes:**

P-values in bold are indicated in Fig 2A or are referred to in the text

| | L262P/L262Pγ #1 + succinate |
|---|---|
| **L262P/L262Pγ #1 + succinate + azide** | **0.2017** |
| **L262P/L262Pγ #2 + succinate + azide** | 0.9605 |

| | L262P/L262Pγ #1 + succinate |
|---|---|
| **L262P/L262Pγ #1 + succinate + pyruvate** | **0.8543** |
| **L262P/L262Pγ #2 + succinate + pyruvate** | 0.0081 |
| **L262P/WTγ + succinate + pyruvate** | <0.0001 |

| L262P/L262Pγ #2 + succinate |
| ---: |
| 0.0019 |
| **0.3733** |

| L262P/L262Pγ #2 + succinate | L262P/WTγ + succinate |
| ---: | ---: |
| >0.9999 | <0.0001 |
| **0.159** | 0.0162 |
| 0.1787 | **0.4167** |
