# Supplementary material for: Oxidative Phosphorylation Is Required for Powering Motility and Development of the Sleeping Sickness Parasite Trypanosoma brucei in the Tsetse Fly Vector
Source: mBio. 2022 Jan 11;13(1):e02357-21. doi: 10.1128/mbio.02357-21 (PMC8749461; doi:10.1128/mbio.02357-21)
Supplement: FIG S4 [file mbio.02357-21-sf004.pdf]

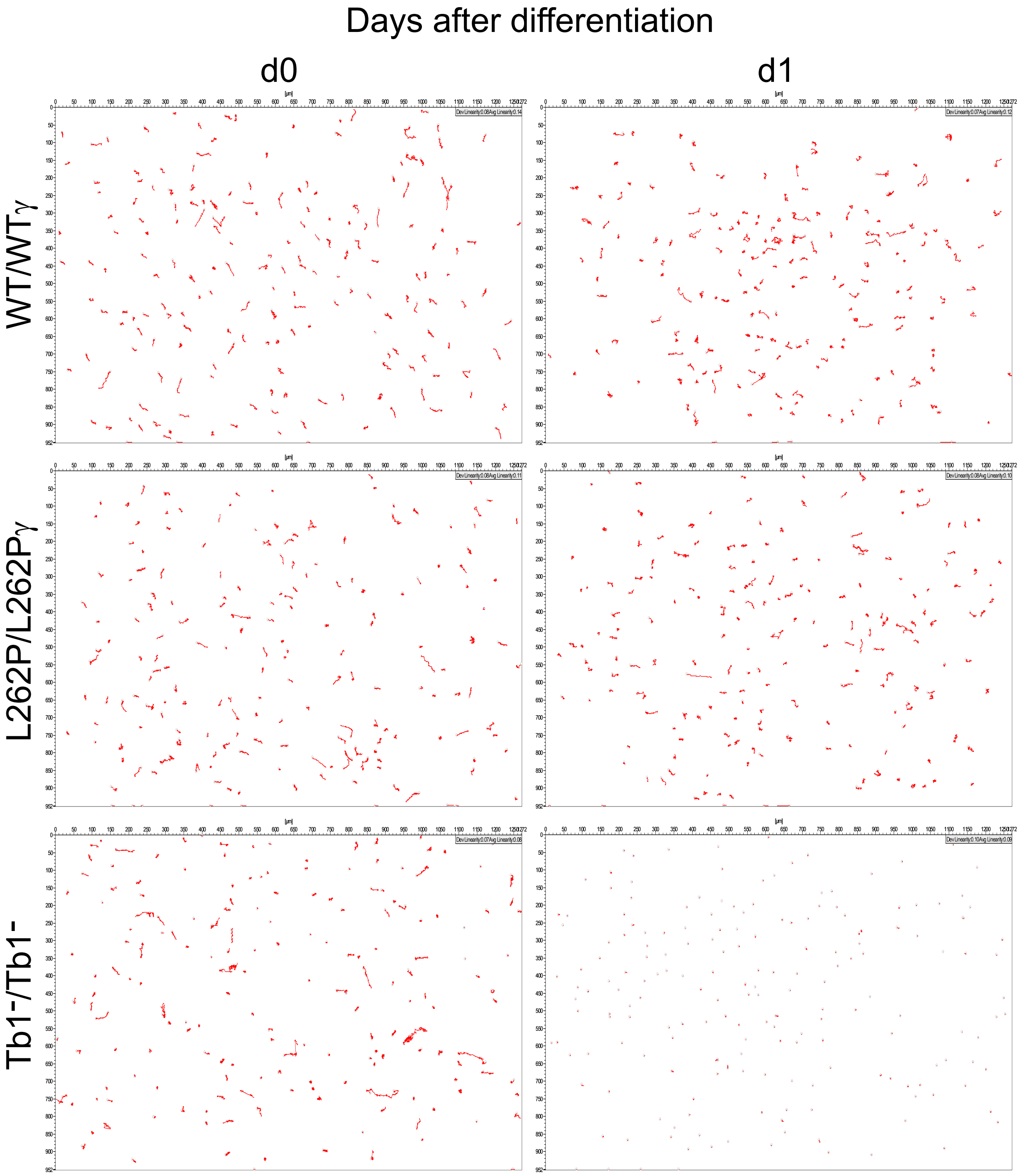

Fig S4. Motility analysis of *in vitro* cultured cells. Representative cell movement tracks taken from videos of differentiated PCF cells *in vitro*. Videos were taken of populations of newly differentiated PCF *T. brucei* at days 0 (d0) and 1 (d1) post differentiation, where d0 is defined as the timepoint after 24 h of exposure to 6 mM CA and 27°C and directly after transfer into SDM80 medium.
